# HIFI: estimating DNA-DNA interaction frequency from Hi-C data at restriction-fragment resolution

**DOI:** 10.1186/s13059-019-1913-y

**Published:** 2020-01-14

**Authors:** Christopher JF Cameron, Josée Dostie, Mathieu Blanchette

**Affiliations:** 1 School of Computer Science, McGill University, Montreal, Canada; 2 Department of Biochemistry and Goodman Cancer Research Center, McGill University, Montreal, Canada

**Keywords:** Chromosome conformation capture, Hi-C, 5C, ChIA-PET, Topologically associating domains, subTADs, Density estimation, Markov random field

## Abstract

Hi-C is a popular technique to map three-dimensional chromosome conformation. In principle, Hi-C’s resolution is only limited by the size of restriction fragments. However, insufficient sequencing depth forces researchers to artificially reduce the resolution of Hi-C matrices at a loss of biological interpretability. We present the Hi-C Interaction Frequency Inference (HIFI) algorithms that accurately estimate restriction-fragment resolution Hi-C matrices by exploiting dependencies between neighboring fragments. Cross-validation experiments and comparisons to 5C data and known regulatory interactions demonstrate HIFI’s superiority to existing approaches. In addition, HIFI’s restriction-fragment resolution reveals a new role for active regulatory regions in structuring topologically associating domains.

## Background

Cells are complex, dynamic environments that require constant regulation of their genes to ensure survival. The advent of chromosome conformation capture (3C) technologies [1], and recent advances in imaging techniques [2], have led to an improved understanding of genome organization and its role in gene regulation [3, 4]. Hi-C [5], a high-throughput derivative of 3C, provides an unparalleled view of three-dimensional (3D) genome organization by capturing all DNA-DNA contacts found within a population of cells. Hi-C has revealed different levels of genome organization, including the topologically associating domains (TADs [6, 7], subTADs [8, 9]), and chromatin compartments [5]. Yet, the potential for a more refined understanding of 3D genome organization remains largely untapped [10].

In a Hi-C experiment, cross-linked chromatin is digested into fragments using a restriction enzyme (RE). Restriction fragments (RF) are then proximity-ligated to obtain a library of chimeric circular DNA. Paired-end sequencing and mapping of reads to a reference genome identifies interacting RFs and their frequency count. The data is conventionally stored as a pairwise read count matrix, RC, where RC_*i*,*j*_ is the number of observed interactions (read-pair count) between genomic regions *i* and *j*. Despite the great sequencing depth of typical Hi-C experiments (200–500 million read pairs), RF resolution *RC* matrices are extremely sparse, with most RF pairs being observed either zero or one time. This sparsity makes measurements of individual interaction frequencies (IFs) between RF pairs inherently stochastic and unreliable. Increasing sequencing coverage is a partial solution, but without improved bioinformatics analyses, the depth of sequencing needed to make reliable estimates of IFs for individual RFs is unmanageable. For this reason, Hi-C data is rarely studied at RF resolution, but instead binned at fixed intervals (e.g., every 25 kb). Unfortunately, reducing the resolution of a Hi-C IF matrix leads to difficulties in studying the interactions between fine-scale genomic elements such as promoters and enhancers.

To improve the resolution of Hi-C data, recent protocols suggest digesting DNA more finely, either with a 4-cutter RE [10, 11] or DNAse I [12], followed by binning at 1 to 5 kb. While these methodologies increase the resolution of a Hi-C IF matrix, they actually worsen the problem of sparsity and stochastic noise. For example, using a 4-cutter RE instead of a 6-cutter results in a 16-fold increase in the number of RFs and a 256-fold increase in RF pairs. This problem can be alleviated by using DNA capture technologies to concentrate sequencing on a predefined set of loci [13, 14], but this approach loses the ability to interrogate the whole-genome conformation in a hypothesis-free manner. Instead, new bioinformatics approaches have been proposed to detect individual significant contacts at high resolution from Hi-C data [15, 16], and a machine learning method has been introduced to smooth Hi-C matrices at 10-kb resolution [17]. Dynamic binning was also proposed as a way to adjust bin size to ensure even read coverage across the genome, enabling locally higher resolution [18]. However, no approach currently exists to obtain complete and accurate IF matrices at RF resolution. Such an approach would be valuable as it would allow researchers to revisit existing datasets and get more information out of them without having to change experimental protocols or generate more experimental data.

Here, we introduce the Hi-C Interaction Frequency Inference (HIFI) algorithms, a family of computational approaches that provide reliable estimates of IFs at RF resolution. HIFI algorithms reduce stochastic noise, while retaining the highest possible resolution, by taking advantage of dependencies between neighboring RFs. We validate these algorithms via cross-validation and a comparison to observations made by independent chromosome conformation assays. We further demonstrate that HIFI improves the detection of contacts between promoters and enhancers. Finally, we illustrate additional benefits of high-resolution Hi-C data analysis by using it to study how active regulatory regions are involved in structuring TADs and subTADs.

## Results

HIFI algorithms aim to reliably estimate Hi-C contact frequencies between all intra-chromosomal pairs of restriction fragments. The output of a HIFI algorithm is an IF matrix per chromosome, where each entry (*i*,*j*) corresponds to the IF of RFs *i* and *j*. As REs do not digest DNA uniformly along the genome, different rows/columns correspond to regions of different sizes. Depending on the RE used, the achievable resolution of Hi-C ranges on average from 434 bp (for a four-cutter such as MboI) to 3.7 kb (for a six-cutter such as HindIII). The high-resolution analysis of Hi-C data faces multiple challenges, of which the sparsity of the observed read-pair data is the most significant. For example, a Hi-C experiment with a very high sequencing depth of one billion read pairs will yield on average approximately 0.1 read pairs per intrachromosomal matrix entry for a six-cutter RE and less than 0.001 for a four-cutter RE. Even at a relatively short distance of 100 kb, the observed number of read pairs for a four-cutter RE almost never exceeds 1. This sparsity results in the observed read-pair count for a given RF pair being a poor (high-variance) estimator of the true IF, except for rare RF pairs located in regions of the Hi-C contact map where IF values are extremely high. All existing solutions to this problem, including the methods introduced in this paper, take advantage of the fact that IFs of neighboring entries in the IF matrix are strongly correlated. In particular, the most common approach to the resolution/accuracy trade-off is to artificially reduce the resolution by binning the raw data to fixed-size intervals (e.g., 25-kb bins). This lower resolution increases the number of reads per bin pair, and thus allows for a more reliable estimation of IF, but at the cost of a loss in biological interpretability. Importantly, no unique bin size is uniformly ideal for an entire IF matrix. Portions of an IF matrix where high IFs are present could support a high-resolution analysis, whereas others, corresponding to lower IF values, may require larger bins for accurate IF estimation.

More specifically, the problem addressed here is the following: consider a Hi-C dataset *H* produced with a given restriction enzyme *e*. For a given chromosome, the raw outcome is stored in an *n*×*n* intrachromosomal matrix *RC*, where *n* is the number of RFs produced by *e*, and RC_*i*,*j*_ contains the number of read pairs mapped to RF pair (*i*,*j*). Our goal is to estimate as accurately as possible the true RF-level interaction frequency matrix, I*F*_*true*_, which is the theoretical *n*×*n* IF matrix one would obtain if one were to sequence an infinitely large version of *H* to infinite depth (scaled for the total number of read pairs). I*F*_*true*_ is affected by a number of library, sequencing, and mapping biases that would need to be corrected in order to allow for proper biological interpretation; many such normalization techniques already exist for this task [19–21]. Our goal here is not to improve upon these techniques, but to work upstream and provide the most accurate estimate of I*F*_*true*_.

Four approaches are introduced and assessed (see the “Methods” section for details), each taking as input matrix *RC* and producing as output an estimate of I*F*_*true*_:

1. The commonly used fixed-binning approach, where the genome is first partitioned into bins containing a fixed number of kilobase (or, alternatively, a fixed number of RFs) and the estimated IF for a given bin pair is the total number of reads whose end points fall within that pair of genomic intervals.
2. A simple kernel density estimation (HIFI-KDE) approach, where the IF estimate at a given matrix entry is obtained as the average of surrounding entries, weighted using a two-dimensional Gaussian distribution with a fixed standard deviation (bandwidth).
3. An adaptive kernel density estimation (HIFI-AKDE) approach, where the bandwidth is chosen dynamically for each matrix entry in order to ensure that a sufficient number of read pairs is available for reliable IF estimation, while maximizing the resolution.
4. An approach based on Markov random fields (HIFI-MRF) where dependencies between neighboring cells are modeled and used to identify the maximum *a* *posteriori* estimate of I*F*_*true*_.

Assessing the accuracy of high-resolution IF inference algorithms is challenging because I*F*_*true*_ is unknown, as Hi-C datasets of infinite sequencing depths are not achievable. Instead, we consider two surrogates. First, we use a cross-validation approach from existing Hi-C data. Second, we assess the predictions against data produced by Chromosome Conformation Capture Carbon Copy (5C [22]), a targeted amplification protocol that achieves a much higher read count per RF pair compared to Hi-C.

### Cross-validation of HIFI algorithms

**Fig. 1:**
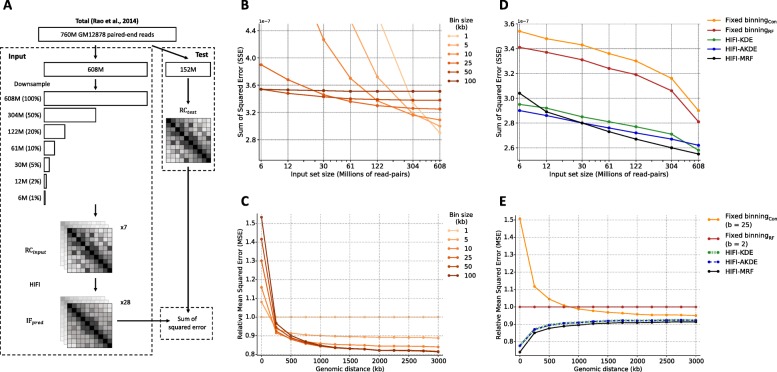
Cross-validation of fixed-binning and HIFI methodologies. **a** Schematic representation of cross-validation methodology to assess the accuracy of fixed-binning and proposed HIFI methodologies. **b** Cross-validation error for canonical fixed-binning approaches, for different bin sizes, as a function of coverage, see also Additional file 1: Figure S1 for similar analyses of RF fixed binning, HIFI-KDE, and HIFI-AKDE. **c** Analysis of canonical fixed-binning error (relative to error with one RF per bin) across genomic distance between RF-pairs. No singular bin size performs best for all genomic distances. **d** Comparison of errors for different approaches. For fixed binning and HIFI-KDE, the optimal bin size or bandwidth was chosen separately for each coverage level. Nonetheless, HIFI-MRF outperforms all other approaches. **e** Comparison of errors (relative to error obtained with fixed binning using two RFs per bin) by genomic distance of RF pairs, using as input a set of 304M read pairs (50% of total training set). HIFI-MRF performs best across all distances

We used cross-validation to assess the accuracy of HIFI algorithms genome-wide. Here, a Hi-C read-pair dataset of high sequencing depth produced by Rao et al. [23] from GM12878 cells using HindIII was first filtered to retain only high-confidence intrachromosomal read pairs. These read pairs were then randomly partitioned into an input set (containing 80% of the set of filtered read pairs, or 607,587,043 read pairs) and a test set (20%, or 151,979,454 read pairs) (Fig. 1a). The input set is then further downsampled into 7 subsets ranging in size from 1 to 100% of the full input set. Mapping and tabulating read pairs at RF-level resolution yields a family of read count matrices: $\mathrm {RC_{input\_ 1}}$, $\mathrm {RC_{input\_ 2}}$, …, $\mathrm {RC_{input\_ 100}}$, and R*C*_*test*_.

Each of the four inference algorithms are evaluated by their application to each of the downsampled input matrices to obtain a predicted IF matrix, I*F*_*pred*_, which is then compared to the test matrix R*C*_*test*_ to obtain the sum of squared errors:

$$ \text{SSE}(\text{IF}_{\text{pred}}, \text{RC}_{test}) = \sum_{i< j}(\text{IF}_{\text{pred}_{{i, j}}} - {\text{RC}}_{\text{test}_{{i,j}}})^{2}   $$

Although R*C*_*test*_ is clearly not equal to I*F*_*true*_, because R*C*_*train*_ and R*C*_*test*_ are sampled independently, the inference approach that minimizes SSE(I*F*_*pred*_,R*C*_*test*_) is also the one that minimizes SSE(I*F*_*pred*_,I*F*_*true*_), and hence, this serves as a valid basis for comparison.

Figure 1b and Additional file 1: Figure S1A show that the accuracy of fixed-binning strategies improves with input set size, and that the optimal accuracy is obtained at different bin sizes for different input set sizes: large bins are ideal for low-coverage training data, whereas smaller bins are better with high-coverage data. More importantly, the fact that read pairs are highly non-uniformly distributed in RC matrices means that the ideal bin size differs depending on the local RC density. In particular, short-range contacts, which typically have higher RC values, can support high-resolution analyses (smaller bins), but those at longer ranges are best estimated with larger bins (Fig. 1c and also Additional file 1: Figure S1B for similar analyses for binning based on number of RFs rather than sequence size). The HIFI-KDE approach with a fixed bandwidth generally obtains better results (Fig. 1d and Additional file 1: Figure S1C), but suffers from the same type of problem, where optimal results are obtained with large bandwidth values for low-coverage regions and lower bandwidth values for high coverage regions. The HIFI-AKDE approach, where different bandwidth values are chosen at each cell based on the surrounding signal density, outperform the first two approaches (Fig. 1d and Additional file 1: Figure S1D), with optimal performance obtained using a *MinimumCount* value of 100 (see the “Methods” section) throughout various coverage levels. HIFI-MRF performs the best overall (Fig. 1d and Additional file 1: Figure S1E), except at extremely low sequencing depths (i.e., 6–12M read pairs). Indeed, for typical sequencing depths (100–250M read pairs), HIFI-MRF improves IF estimation accuracy over the entire range of genomic distances (Fig. 1e) producing estimates that are 5–40% more accurate than those obtained by fixed-binning approaches and 5% more accurate than HIFI-KDE and HIFI-AKDE, see Additional file 1: Figure S2 for an example of a HIFI-MRF-processed HiC matrix and comparison to fixed-binning analysis. We attempted to use the same strategy to evaluate HiCPlus [17], a machine learning technique for high-resolution analysis of Hi-C data, but found that the model did not perform well on non-bias-corrected Hi-C data for this analysis. Finally, to further demonstrate the robustness of HIFI-MRF’s IF estimates, 2 replicates of the mouse embryonic stem cell (mESC) Hi-C data produced by Bonev et al. (2017) [24] were processed separately. HIFI-MRF produced contact maps that yielded improved correlations between replicates when compared to fixed-binning approaches (Additional file 1: Figure S3).

### Validation against 5C data

**Fig. 2:**
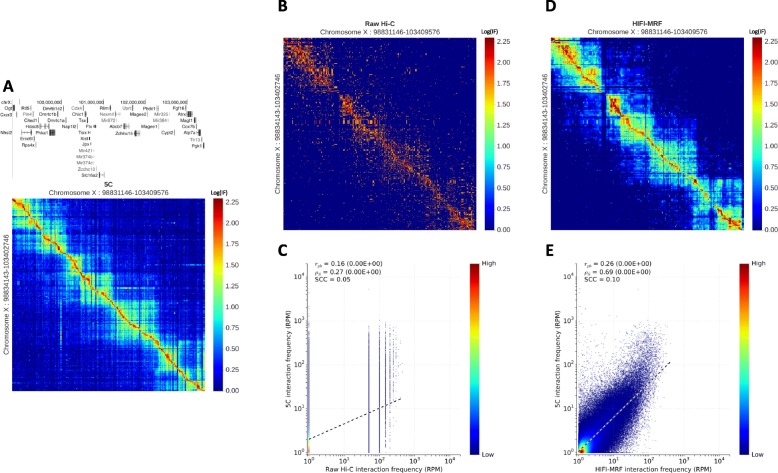
Recapitulation of 5C observations by HIFI-MRF. **a** IF matrix obtained by 5C of the 4.5-Mb locus surrounding the *Xist* gene in mouse embryonic stem cells [7]. Note the use of true-size heatmaps, where the height (resp. width) of a row (resp. column) is proportional to the size of the RF it represents. **b** Raw, RF resolution Hi-C data for the same region [6]. **c** Correlation of 5C and raw Hi-C data at RF resolution (Pearson *r*_*pb*_ = 0.16, *p* value <10^−16^; Spearman *ρ*_*s*_ = 0.27, two-sided Student’s *t* test *p* value <10^−16^); stratum-adjusted correlation coefficient (SCC) [30] = 0.05). **d** IF matrix estimated by HIFI-MRF from the same Hi-C data. Observe the similarity to the 5C data in **a**. **e** Correlation of 5C and HIFI-MRF-processed Hi-C data at RF resolution (Pearson *r*_*pb*_ = 0.26, *p* value <10^−16^; Spearman *ρ*_*s*_ = 0.69, *p* value <10^−16^); SCC = 0.10)

5C has been used to study the conformation of moderate-size genomic regions (100 kb-5 Mb), including the beta-globin locus [22, 25], the *HOX* clusters [8, 26, 27], the *CFTR* locus [28, 29], and the *Xist* locus [7]. 5C allows for a high sequencing depth measurement of the IF of each RF pair within given genomic regions, which improves the accuracy of RF-level IF estimates. As such, 5C data constitutes an excellent benchmark to compare different inference approaches. We analyzed data from two cell types for which both 5C and Hi-C data are available: (i) a 4-Mb region around the *Xist* gene (Fig. 2a, b) in mouse embryonic stem cells (mESC; Hi-C data from Dixon et al. [6]; 5C data from Nora et al. [7]), and (ii) a 2.7-Mb region around the *CFTR* gene (Additional file 1: Figure S4A,B) in human GM12878 cells (5C data from Smith et al. [29]; Hi-C data from Rao et al. [23]). In the GM12878 dataset, which has higher Hi-C sequencing depth (760M mapped read pairs genome-wide), the correlation between raw Hi-C and 5C data is moderate (Spearman *ρ*_*s*_ = 0.45; Additional file 1: Figure S4C), but it is improved by the application of HIFI-MRF (*ρ*_*s*_ = 0.71; Additional file 1: Figures S4D,E and S5). In the mESC dataset, with lower Hi-C sequencing coverage (122M read pairs), the correlation of raw 5C against raw Hi-C data is relatively weak (*ρ*_*s*_ = 0.27; Fig. 2c) but improves to nearly the same level as in the first dataset from the application of HIFI-MRF (*ρ*_*s*_ = 0.69; Fig. 2d, e and Additional file 1: Figure S6). Strata-adjusted correlation coefficients (SCC) [30], which factor out correlations induced by genomic distance dependencies, are also improved by the application of HIFI-MRF. Also note how, in both cases, the intricate structure of TADs, as well as some of the finer looping events, become apparent in the HIFI-MRF-processed Hi-C data (Fig. 2d and Additional file 1: Figure S4D). The accuracy of HIFI-MRF estimates was found to be largely independent of the fragment bias correction algorithm applied, with the four normalization algorithms implemented in Juicer [31] yielding similar results (see Additional file 1: Figure S7).

**Fig. 3:**
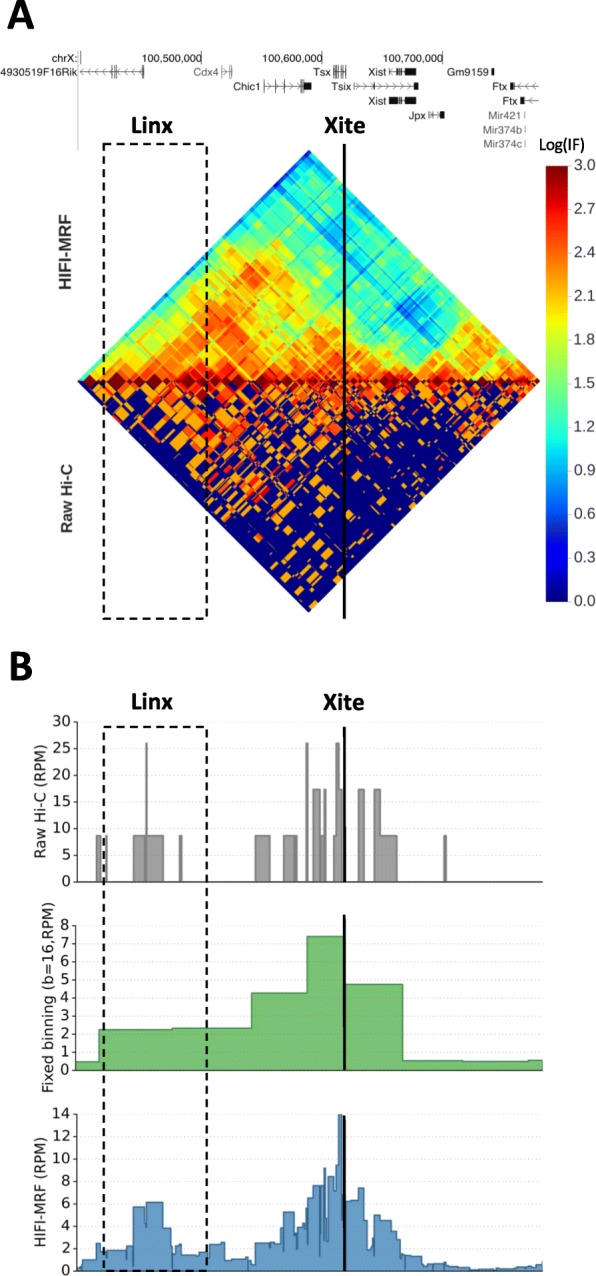
HIFI-MRF reveals fine-scale regulatory contacts in Hi-C data. Heatmap (**a**) and virtual 4C [60, 61] plots based on raw, binned, or HIFI-MRF processed data (**b**) showing the long-range interaction between *Tsix* and its transcriptional regulator, *Linx*, on chromosome X of female mice as observed by Nora et al. [7] using 5C. This interaction is more easily observed in HIFI-MRF data than in raw or binned Hi-C data

Indeed, the application of HIFI-MRF to Hi-C data allows for the detection of regulatory contacts that could previously only be observed using 5C. For example, Nora et al. [7] used 5C to observe a long-range interaction between *Tsix* and its transcriptional regulator—a large intervening non-coding RNA called “Linx”—occurring in female mice as a component of X inactivation. This interaction is very clearly observed in the HIFI-MRF-processed Hi-C data (Dixon et al. [6]), whereas it is difficult to distinguish from background in raw or binned Hi-C data (Fig. 3a, b). These results demonstrate that HIFI-MRF can be used to analyze existing Hi-C datasets and potentially lead to novel discoveries at finer genomic scales.

### Validation against externally predicted chromatin contacts

**Fig. 4:**
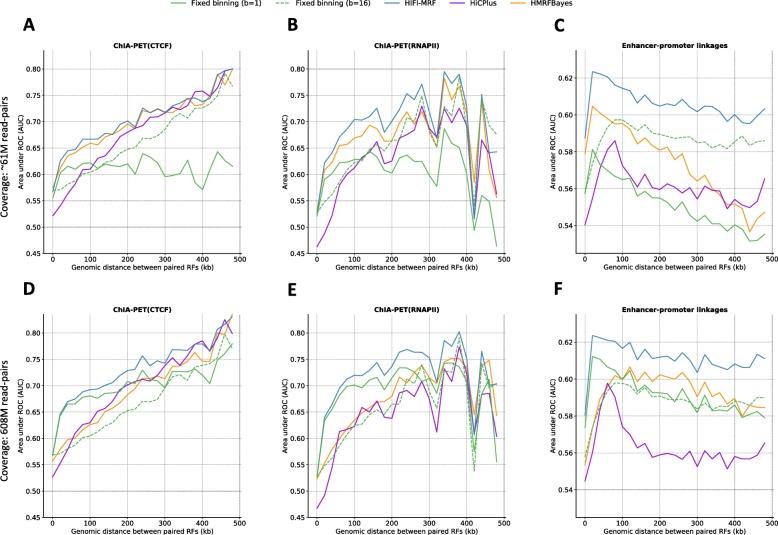
Positive/negative RF contact delineation analysis. The ability of different HiC data analysis approaches to distinguish positive from negative (control) contacts is measured, for various data sets, using the area under the receiver operating characteristic curve (AUROC) for univariate predictors using as input the predicted IF values. **a**, **d** CTCF-mediated contacts identified by ChIA-PET [33]. **b**, **e** RNAPII-mediated contacts identified by ChIA-PET [33], **c**, **f** Inferred enhancer-promoter linkages based on DHS correlation [35]. To allow for the comparison with HiCPlus and HMRFBayes, only contacts occurring on chromosomes 9 to 22, X, and Y, and within a distance of 1 Mb, are analyzed. Top (**a**–**c**) and bottom (**d**–**f**) rows represent the performance of the classifiers applied to Hi-C data of size 60.8M (10% of input set) and 608M (100% of input set), respectively. Genome-wide results for HIFI are shown in Additional file 1: Figure S8. Similar results are observed for ChIA-PET RAD21 (Additional file 1: Figure S9)

To more fully assess the extent to which HIFI-MRF-processed Hi-C data can be used to identify biologically relevant contacts, we asked whether it can also confirm chromatin interactions found through alternative approaches. Specifically, we considered a set of contacts identified by chromatin interaction analysis with paired-end tag sequencing (ChIA-PET [32]) in GM12878 cells, bound either by CTCF (92,114 contacts [33]), RNA polymerase II (PolII—192,394 contacts [33]), or RAD21 (38,952 contacts [34]). A set of computationally inferred contacts identified by correlation of DNAse I hypersensitivity signals across multiple cell types [35], as well as cohesin-mediated interactions in GM12878 captured by Hi-C chromatin immunoprecipitation (HiChIP [36] — 10,254 contacts), were also considered. For each set of contacts, a set of negative (control) fragment pairs were chosen by randomly re-pairing the same RFs. We then measured, for each range of genomic distance, the extent to which positive contacts could be distinguished from negative contacts on the basis of normalized HIFI-MRF Hi-C data, by measuring the area under the receiver operating characteristic curve (AUROC) of a univariate predictor using the RF pair’s inferred IF value as a predictive variable. Higher AUROC values indicate improved ability to distinguish positive from negative contacts. We observe that HIFI-MRF-processed Hi-C data allows significantly better detection of validated contacts compared to fixed-binning approaches, for all 5 datasets, across all genomic distance ranges, and both at low (61M read pairs obtained by downsampling; Fig. 4a–c and Additional file 1: Figures S8A-C, S9A,B, S10A,B) and high (608M read pairs; Fig. 4d–f and Additional file 1: Figures S8D-F, S9C,D, S10C,D) sequencing depths. Notably, the ability to distinguish positive from negative ChIA-PET contacts is relatively poor at short distances (< 50 kb) because nearly all pairs have very high IF values, but improves considerably at longer range (300–500 kb). In contrast, contacts inferred based on DHS correlations are more difficult to identify overall (AUROC < 0.6), becoming increasingly so at longer ranges. We speculate that this loss in detection power may be due to an increased error rate present in this benchmark dataset. Remarkably, the application of HIFI-MRF to low-coverage Hi-C data yields predictive power that is nearly as good as in the high-coverage dataset (compare panels Fig. 4a–c to 4d–f), suggesting that HIFI-MRF is able to identify functional contacts even in Hi-C data of moderate depth. Figure 4 also includes the results for HiCPlus [17] and HMRFBayes [15], an approach for the detection of significant contacts at RF resolution (see the “Methods” section). Overall, HIFI-MRF clearly outperforms these two approaches, although HMRFBayes performs nearly equally well for some low-coverage datasets (Fig. 4a–c). The advantage of HIFI-MRF is particularly noticeable at short- to medium-range distances (< 200 kb). Taken together, these results show that using HIFI-MRF to process Hi-C data improves the ability to delineate individual chromatin contacts.

### HIFI allows new insight into fine-level genome organization

**Fig. 5:**
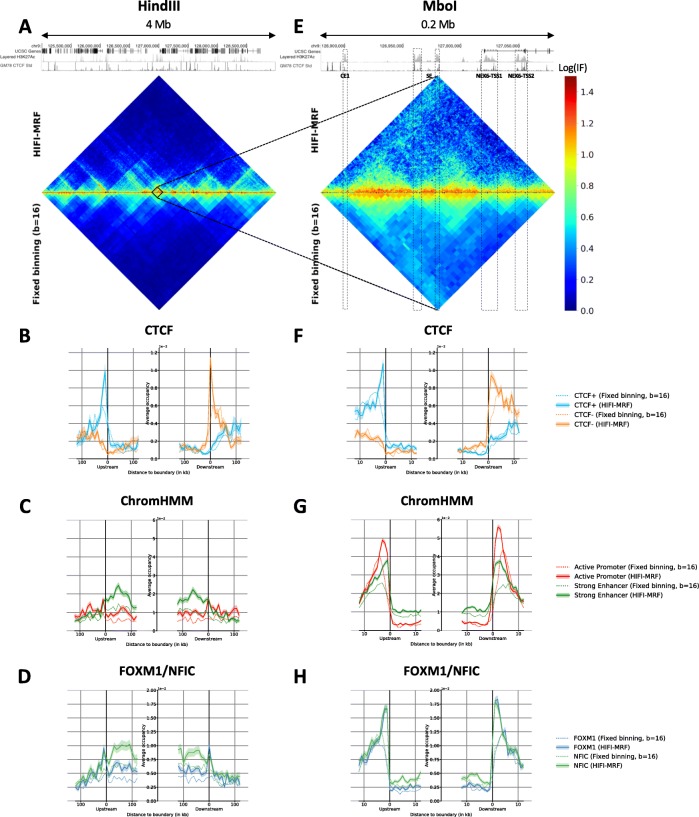
Analysis of RF resolution TAD and subTAD boundaries in GM12878. Analyses were performed on both Hi-C data resulting from a HindIII (3.4 kb per RF on average (**a**–**d**)) and a MboI restriction digest (434 bp per RF on average (**e**–**h**), from Rao et al. [23]). TAD and subTAD boundary predictions were made on IF matrices produced either by HIFI-MRF or a fixed-binning approach (16 RF per bin, i.e., approx. 50 kb per bin for HindIII and 7 kb per bin for MboI). **a** IF matrices produced by HIFI-MRF (top) and fixed binning (bottom) for a 4-Mb locus surrounding the NEK6 locus (chr9:124999244-128993971). **b**, **f** CTCF occupancy as a function of distance to the nearest TAD (**b**) or subTAD (**f**) boundary, separately for sites on the forward and reverse strands. Convergent CTCF sites are enriched at both TAD and subTAD boundaries. Shaded band indicate 95% confidence intervals of the estimate of the mean occupancy. **c**, **g** Coverage of active promoters (red) and strong enhancers (green) identified by ChromHMM, as a function of the distance to the nearest TAD (**c**) or subTAD (**g**) boundary. These regions are very strongly enriched just outside of subTAD boundaries, but less so around TAD boundaries. **d**, **h** Occupancy of two transcription factors, FOXM1 and NFIC, as a function of distance to the nearest TAD (**b**) or subTAD (**f**) boundary. While most TFs have an occupancy peak at TAD and subTAD boundaries, the extent of the enrichment within TADs varies from low (e.g., FOXM1) to high (e.g., NFIC). **e** IF matrices produced by HIFI-MRF (top) and fixed binning (bottom) for the 200-kb NEK6 locus (chr9:126879748-127079891). Regulatory regions identified in Huang et al. [62] are marked SE (super enhancer), CE1 (conventional enhancer), and NEK6-TSS1 and NEK6-TSS2 (alternative promoters). Notice how all these regions lie between visible subTADs

The high accuracy and resolution afforded by HIFI enables researchers to answer questions that are difficult to address with lower-resolution analyses of Hi-C data. Here, we illustrate one such application: the high-resolution analysis of TAD and subTAD boundaries. We used a modified directionality index (DI) score, originally introduced by Dixon et al. [6] (see the “Methods” section), to identify 5000 TAD boundaries in the HindIII-GM12878 Hi-C data. Boundary predictions were performed at two resolutions: (i) RF resolution using HIFI-MRF-processed data (3.7 kb on average; Fig. 5a, top heatmap) and (ii) classical fixed-binning approach (16 RF ≈ 50 kb per bin; Fig. 5a, bottom heatmap). Using ENCODE ChIP-seq datasets [37], we quantified the occupancy of DNA-binding proteins relative to TAD boundaries. Consistent with previously reported observations and models [23, 38, 39], CTCF (Fig. 5b) showed a remarkable enrichment immediately outside of these boundaries, with sites on the plus strand sharply peaking at upstream TAD boundaries and those on the minus strand peaking at downstream boundaries. Similar enrichments at TAD boundaries are observed for RAD21, SMC3 (cohesin complex), YY1, and ZNF143 (Additional file 1: Figure S12), consistent with previous reports [6, 40–45]. Although the same phenomenon is visible in fixed-binning data, the peaks are much sharper (narrower and higher) in HIFI-MRF data, indicating that RF resolution allows more accurate calls of TAD boundaries.

We next studied the role of TAD boundaries in gene regulation, by looking at the distribution of active regulatory regions, as annotated by ChromHMM [46] based on cell type-specific histone marks and DNA accessibility data. We observe a moderate enrichment for active promoters immediately outside TAD boundaries (only visible in HIFI-MRF processed data) and for strong enhancers within TADs. This trend is partially reflected in the occupancy profiles of several transcription factors (Fig. 5d and Additional file 1: Figure S13). These transcription factors (in particular EBF1, EP300, IKZF1, MEF2A, MEF2C, and NFIC) exhibit a gradual enrichment toward the middle of TADs, together with a small but well-defined, CTCF-like peak just outside TAD boundaries. Some (e.g., FOXM1, IRF4, and RUNX3) show a more prominent peak at TAD boundaries (Additional file 1: Figure S14A-C), while others (e.g., GABPA, MYC, and SIX5) demonstrate a depletion of occupancy within TADs (Additional file 1: Figure S14D-F). Notice that in many cases, the enrichment at TAD boundaries is only apparent based on HIFI-MRF data and would likely be missed using data binned at 50 kb resolution.

We then repeated the analysis (HIFI-MRF followed by TAD boundary calls) on Hi-C data generated on the same cell line using the 4-cutter MboI restriction enzyme, with cut sites every 434 bp on average. Due to the size of the 4-cutter IF matrices involved, analyses were limited to RF pairs within a maximum distance of 1 Mb. The extremely high resolution of this dataset (Fig. 5e) provides opportunities to study fine structures such as subTADs [8, 9], which are difficult to study at lower resolutions. We used the HIFI-MRF MboI-GM12878 data, and the same modified DI approach to identify a set of 25,000 domain boundaries, of which approximately 2500 matched a HindIII-GM12878 TAD boundary (within 25 kb). The remaining ∼ 22,500 boundaries are not detected in the HindIII data and likely correspond to subTAD boundaries. Repeating the occupancy analysis against subTAD boundaries, the same enrichment for convergent CTCF sites is observed (Fig. 5f), but a very different picture emerges with respect to regulatory regions. Most notably, active promoters, and to a lesser extent strong enhancers, have a clear tendency to occupy regions that lie immediately outside subTADs (Fig. 5g; see also example in Fig. 5e). Indeed, the density of active promoters is approximately 30 times higher in the 1-kb region that precedes a subTAD boundary than in the 1-kb region that follows one. A similar enrichment is found in the inter-subTAD regions for FOXM1 and NFIC (Fig. 5h), and nearly all transcription factors studied. These results are consistent with a model where active regulatory regions play a key role in partitioning TADs into subTADs.

## Discussion and conclusions

Hi-C has become a commonly used approach to map 3D chromatin organization genome-wide. Since its introduction in 2009, the method has been updated many times to improve upon accuracy and resolution, or to target specific types of contacts. However, to date, using Hi-C data to accurately and systematically identify fine-scale chromosome contacts remains challenging, mostly because the sequencing depth required to achieve high-resolution contact maps is too great. To overcome the sparsity of contact information and increase the signal-to-noise ratio, Hi-C data is traditionally binned at fixed intervals along chromosomes to produce lower-resolution matrices [10]. This lower-resolution representation of Hi-C data limits its application in studies of genomic regulatory networks or mechanisms of disease, which require robust, high-resolution 3D genomics data.

Here, we introduced HIFI, a family of density estimation algorithms that allow for the observation of high-resolution (at the restriction-fragment scale) genomic contacts from Hi-C data of various sequencing depths. Our results show that HIFI algorithms, and in particular those based on Markov random fields (HIFI-MRF), provide highly accurate estimates of Hi-C interaction frequency at RF resolution and outperform classical fixed-binning approaches. We demonstrate that HIFI-MRF recapitulates contact data obtained by 5C and also captures interactions detected by ChIA-PET (Fig. 4) better than HiCPlus and HMRFBayes [15]. Unlike the former, HIFI is easy to use and does not require special equipment (GPUs) to run within a reasonable time frame. HIFI also runs more than 100 times faster than the HMRFBayes. The high resolution and accuracy provided by HIFI allows analyses and discoveries that could not be made with lower-resolution Hi-C data. For example, HIFI allows for the identification of TAD boundaries at RF resolution, which provides a unique opportunity to finely delineate the role of different DNA-binding proteins. Benefiting from the RF resolution achieved with HIFI-MRF, we show that CTCF, RAD21, SMC3, and ZNF143 are enriched just outside both TAD and subTAD boundaries, and their sharp depletion within TADs may be a major contributor to the formation of TAD boundaries (Fig. 5). In addition, we detail a set of transcription factors (based on ENCODE ChIP-seq data) that are found to be enriched at RFs labeled as TAD boundaries (Fig. 5b, c). Finally, we highlight the new observation that active enhancers and promoters appear to provide structure to TADs, whereby DNA located between consecutive active regulatory regions form subTADs. This is obviously just an illustration of insights that can be gained from the analysis of Hi-C at high resolution. Others would include the use of HIFI-processed Hi-C data to further dissect the mechanisms of genome organization and to prioritize non-coding variants obtained from genome-wide association (GWAS) or expression quantitative trait loci (eQTL) studies, as is starting to be done with capture Hi-C data [47].

Our work also addresses the extent to which the accuracy of IF estimates degrade in response to lowering sequencing depths of a Hi-C library. The accuracy of HIFI (specifically KDE, AKDE, and MRF algorithms) was shown to reduce gracefully as coverage decreased (see Fig. 1d—where the SSE drops logarithmically to the size of input data). The impact of coverage depth vs. accuracy of IF estimates depends on the biological question at hand. For example, TADs can clearly be identified quite accurately at low sequencing depth (although pinpointing their precise boundaries requires higher depth), while the detection of enhancer-promoter loops may require depths much higher than those made possible by HIFI. The results presented in Fig. 4 and Additional file 1: Figures S8-S10 demonstrate that, using HIFI, a low-coverage Hi-C data set (61M reads) can be used quite effectively to recapitulate ChIA-PET and HiChIP data, obtaining results only 3–5% worse (in terms of AUC) compared to a high-coverage data set (608M reads).

We next studied the extent to which loop calling is facilitated by HIFI. To this end, we developed and incorporated into HIFI a simple loop-calling program based on the algorithm used in HiCCUPS [23]. This program operates directly on HIFI-processed IF matrices rather than canonical binned normalized data, which HiCCUPS may only be applied to. Additional file 1: Figure S11 demonstrates that HIFI-processed Hi-C data results in a higher fraction of predicted loops supported by ChIA-PET data (CTCF and RNAPII) compared to HiCCUPS. This increase is especially notable within a distance range of 250 kb.

Importantly, applying HIFI to a given Hi-C matrix is akin to simulating the sequencing of the same Hi-C library at extremely high depth. Several aspects of the Hi-C experimental protocol may introduce biases in the library, including the choice of restriction enzyme or ligation/fixation approach. In fact, our initial plan was to use Rao et al.’s very deeply sequenced, MboI-digest Hi-C data [23] as a basis to evaluate the processing of their HindIII data for the same cell type GM12878. This plan was thwarted by the relative lack of similarity between the two data sets at high resolution. Nonetheless, while HIFI does not aim to correct these types of biases, it may actually help to reveal and study them.

While HIFI provides a significant improvement over previous methodologies for handling Hi-C matrix sparsity, there remains several directions for possible improvements. First, HIFI is relatively slow, requiring roughly an hour per chromosome (at HindIII resolution), due to the size of the matrices analyzed and the complexity of MRF-based inference. Improved algorithms, multi-threading, and GPU-based computation are expected to provide significant speedups and are under development. These improvements will also allow the calculation of confidence intervals for estimated contacts frequencies, using Markov chain Monte Carlo sampling. Machine learning (ML) approaches, such as convolutional neural nets (CNN), offer an alternative to probabilistic approaches like HIFI-MRF. In recent work by Zhang et al. [17], the authors showed that CNNs can be trained on Hi-C data to increase the resolution from 40 to 10 kb. Being model-free, ML approaches have the potential to discover and take advantage of unsuspected dependencies in the data. However, these models have yet to produce RF resolution data and thus remain limited in their ability to provide biological support as shown in this manuscript. In addition, being intrinsically complex models, prediction errors may occur in unexpected manners.

The impact of fragment-specific biases on RF resolution Hi-C data also deserves further studies [19, 48]. Although HIFI is not by itself a bias correction approach, improvements to RF resolution bias estimation, based for example on the work of Gilgenast and Phillips-Cremins [49], will contribute to improving HIFI’s accuracy.

In conclusion, the HIFI algorithms and software described in this manuscript allow for accurate, high-resolution analyses of 3D genome organization using Hi-C data. RF resolution Hi-C data allows for the recapitulation of observations made by 5C, a better separation of positive and control/background contacts, RF resolution TAD and subTAD boundary calling, and the identification of potential DNA-DNA contacts and TF enrichments that drive changes in chromatin architecture and gene regulation. By operating upstream of many Hi-C data analysis tools (e.g., loop, TAD, and compartment predictors as well as fragment bias normalization), HIFI can easily be inserted in a number of Hi-C data analysis pipelines, and we believe that the research community will be quick to take advantage of this family of new algorithms.

## Methods

### Hi-C read-pair pre-processing

The publicly available Hi-C User Pipeline (HiCUP [50]) v0.5.3 was used to process raw sequencing reads. HiCUP-mapped reads to the human (hg19) genome are also filtered to remove expected artifacts resulting from the sonication and ligation steps (e.g., circularized reads, reads with dangling ends) of the Hi-C protocol. Mapped reads were further filtered for a Mapping Quality Score (MAQ) greater than 30 [19]. BAM/SAM-mapped read files were then converted (by our ’BAMtoSparseMatrix.py’ script) to a raw read-pair count matrix RC, stored using a sparse matrix TSV file format, before use with HIFI.

### HIFI algorithms

The HIFI package is available at https://github.com/BlanchetteLab/HIFI [51] and 10.5281/zenodo.3556842 [52] under the GNU Lesser General Public License. It consists of a C++ program for IF estimation, together with Python scripts for input data formatting and the true-size IF matrix visualization. This section provides algorithmic details.

#### Fragment-specific bias calculation

Factors such as fragment size, GC content, and mappability affect the observed read count matrix RC. For each fragment *i* of chromosome *c*, we estimate this bias as:

$$ \text{bias}_{i}=\frac{\sum_{j}\text{RC}_{i,j}}{\sum_{i^{\prime},j^{\prime}}\text{RC}_{i^{\prime},j^{\prime}}}\cdot n_{\text{fragments}},   $$

where *n*_fragments_ is the number of RFs on that chromosome. Computed biases are used to obtain a normalized read count matrix nRC, where $\text {nRC}_{i,j}=\frac {{\text {RC}}_{i,j}}{\text {bias}_{i}\cdot \text {bias}_{j}}$.

#### Fixed-binning approach

In the fixed-binning approach, the user specifies the value binSize, which is the number of consecutive RFs to be binned together. Defining bin_*i*_={*j*:⌊*j*/binSize⌋=⌊*i*/binSize⌋}, we then obtain the following estimate of interaction frequency for RF pair (*i*,*j*):

$$ \text{IF}^{\text{FB}_{i,j}}=\frac{\sum_{a\in \text{bin}_{i}}\sum_{b\in \text{bin}_{j}}\text{nRC}_{a,b}}{\text{binSize}^{2}}.   $$

#### Fixed kernel density estimation

This approach follows the standard two-dimensional kernel density estimation (KDE) procedure [53, 54], where predicted IF for RF pair (*i*,*j*) is obtained as a weighted sum of the entries of RC surrounding (*i*,*j*), parameterized by bandwidth parameter *h*. Specifically, we set:

$$ \text{IF}^{\text{KDE}_{i,j}} = \frac{\sum_{a=-3h}^{3h}\sum_{b=-3h}^{3h}w(a,b;h)\cdot \text{nRC}_{i+a,j+b}}{\sum_{a=-3h}^{3h}\sum_{b=-3h}^{3h}w(a,b;h)},   $$

where $w(a,b;h)=\frac {e^{-\frac {a^{2}+b^{2}}{2h^{2}}}} {\sqrt {2\pi h^{2}}}$. Near the edges of the matrix, values of *a* and *b* such that indices (*i*+*a*,*j*+*b*) fall outside the matrix are excluded from the sums of both the numerator and denominator.

#### Adaptive kernel density estimation

This approach is similar to the fixed KDE, except that the value of the bandwidth parameter *h* is chosen separately for each pair (*i*,*j*). Specifically, we choose *h*_*i*,*j*_ to be the smallest value such that:

$$ \text{cov}_{i,j}=\sum_{a=-3h}^{3h}\sum_{b=-3h}^{3h}{\text{RC}}_{i+a,j+b}\geq \text{MinimumCount},   $$

where “MinimumCount” is a user-defined parameter (we find the MinimumCount = 100 works well in practice and use this value as the default). In other words, the regions of the matrix that tend to have larger RC values are estimated using smaller bandwidths (i.e., higher resolution), whereas those that are more sparse use larger bandwidths. HIFI-AKDE results in a fine resolution in dense regions and a lower resolution in sparser areas of the matrix. In order to speed up the computation of *h*_*i*,*j*_, we use a pre-computed cumulative matrix, cumRC, where:

$$ \text{cumRC}_{i,j}=\sum_{a=1}^{i}\sum_{b=1}^{j}\text{RC}_{a,b},   $$

which allows the calculation of cov_*i*,*j*_ in constant time:

$$\begin{array}{*{20}l} \text{cov}_{i,j} = & \ \text{cumRC}_{i+3h,j+3h}-\text{cumRC}_{i+3h,j-3h}\\ & -\text{cumRC}_{i-3h,j+3h}+\text{cumRC}_{i-3h,j-3h} \end{array} $$

#### Markov random field estimation

A Markov random field (MRF) describes a set of random variables interconnected via a lattice of dependencies. Let us denote by $\phantom {\dot {i}\!}\text {IF}^{\text {MRF}_{i, j}}$ the latent IF value we aim to estimate at position (*i*,*j*). We model dependencies between the neighboring cells using a log-normal distribution:

1 $$ \log(\text{IF}^{\text{MRF}_{i,j}}) \sim \mathcal{N}\left(\mu=\log(\text{MedNeigh}_{i,j}),\sigma_{i,j}^{2}\right),  $$

where MedNeigh_*i*,*j*_ is the median of the eight IF^MRF^ cells surrounding cell (*i*,*j*). We chose to model this dependency using the median instead of the mean of the neighbors because it allows for sharper transitions regions such as TAD boundaries. The value of $\sigma _{i,j}^{2}$ is set to *α*· logMedNeigh_*i*,*j*_). *α* determines the level of dependency between adjacent cells. A small *α* value results in a predicted IF matrix that is very smooth, whereas larger values result in bumpier or less smooth matrices. The value used here (*α*=0.2) was chosen via a grid search to maximize the likelihood of a left-out subset of the training data (validation set). However, HIFI-MRF is quite insensitive to this hyperparameter as the prior it defines is weak compared to the data itself.

We model the dependency between the observed read count RC_*i*,*j*_ and the estimated true IF value I*F*^*MRF*^_*i*,*j*_ using a Poisson distribution:

$$ \text{RC}_{i,j}\sim \text{Poisson}\left(\lambda=\mathrm{IF^{MRF}}_{i,j}\cdot \text{bias}_{i}\cdot \text{bias}_{j}\right).   $$

HIFI also supports the use of a negative binomial distribution to model RC from IF, to allow for increased dispersal of RC values via a user-defined multiplier of variance. We then seek the matrix I*F*^*MRF*^ that maximizes Pr[RC,I*F*^*MRF*^]= Pr[I*F*^*MRF*^]· Pr[RC∣I*F*^*MRF*^]. We first initialize the I*F*^*MRF*^ matrix using the output of the HIFI-AKDE algorithm. We then optimize I*F*^*MRF*^ using iterated conditional mode (ICM) [55] algorithm. Each iteration involves revising the value of each entry I*F*^*MRF*^_*i*,*j*_ so as to maximize the joint probability of I*F*^*MRF*^ and RC. Revising the value of I*F*^*MRF*^_*i*,*j*_ only alters the probability calculation at position *i*,*j* and the eight neighboring cells (because their median may have changed), and thus probability calculations can be limited to that portion of the matrix. Because of the use of the median (rather than the mean), the joint probability function is not differentiable. Instead, the update to I*F*^*MRF*^_*i*,*j*_ is performed by grid search over a small range of multiplicative factors. Convergence is usually achieved in five to ten iterations over the entire matrix.

Despite using the median rather than the mean to model inter-cell dependencies, some bleed-in effect is observed at TAD boundaries. To prevent those, we designed an approach where the nRC matrix is first scanned to identify sharp horizontal or vertical transitions characteristic of TAD boundaries. Horizontal boundaries are defined by a row index *i* and a pair of column indices *j* and *j*^′^ and will be set if the distribution of nRC values in $\text {nRC}_{i, j\ldots j^{\prime }}$ differs significantly from that in $\text {nRC}_{i+1, j\dots j^{\prime }}$, as determined by a Kolmogorov-Smirnov test. More precisely, boundaries are set greedily, starting with the most significant boundary matrix-wide, and iteratively adding more boundaries, provided they do not overlap previously set boundaries, until the KS statistic falls below a user-defined threshold (the value of 1.5 was used here). Vertical boundaries are symmetrical to horizontal boundaries. Boundaries are then used in the HIFI-MRF model to prevent certain neighbors from contributing to the neighborhood median of a given cell. Specifically, cells (*i*^′^,*j*^′^) that sit on the opposite side of a boundary from cell (*i*,*j*) are excluded from the neighborhood of (*i*,*j*).

#### Output matrices

HIFI can produce either a normalized or non-normalized output. Non-normalized outputs are obtained as $\phantom {\dot {i}\!}\text {IF}^{\text {MRF}_{i, j}} \cdot \text {bias}_{i} \cdot \text {bias}_{j}$. In this manuscript, normalized outputs were used throughout, except for the cross-validation experiment.

#### Conversion between fixed and restriction fragment resolutions

HIFI operates at RF resolution, whereas other approaches operate at fixed resolutions (e.g., 5, 25, or 50 kb). To convert from fixed to RF resolution, we tested three alternatives: the “direct” scheme, which assigns each restriction fragment the frequency of the fixed bin that their 3 ^′^ end resides within; (ii) the “counts” scheme, which divides a fixed bin’s interaction frequency by the number of 3 ^′^ fragment ends found within it and then assigns this value to each of those fragments; (iii) the “weighted” scheme, which determines the proportion of each fragment that overlaps with a given fixed bin, then assigns each fragment its relative proportion of that bin’s interaction frequency. The “direct” conversion scheme was found to be the most robust and perform more consistently across datasets and is the one that was used throughout the paper.

### Alternative approaches

The source code for HiCPlus [17] was obtained from https://github.com/zhangyan32/HiCPlus. Models were trained on Hi-C data from chromosomes 1–8 at 10 kb resolution, within a range of 2 Mb, as recommended. Input and target contact frequencies were obtained from input set and test RC matrices, respectively. Models were provided 100 epochs (10 times more than recommended) to converge while ensuring overfitting did not occur.

HMRFBayes [15] was obtained from http://www.unc.edu/~xuzheng/HMRFHiCFast/tutorial.php. The HMRFBayes program was provided the observed and expected contact frequencies for paired restriction fragments within 1-Mb bins along chromosomes 9-X, where the expected contact frequency was calculated as follows:

$$ \text{Expected}_{i,j} = \frac{\text{TotalReadRow}_{i}\cdot \text{TotalReadColumn}_{j}}{\text{TotalReadPairInMatrix}}   $$

### Chromatin loop calling using HIFI data

Loop calling of HIFI-processed Hi-C data was performed using an algorithm similar to HiCCUPS [23]. We refer the reader to Fig. 3a and Supplementary material section VI.a.3 of Rao et al. [23]. Here, authors of [23] define a distance-normalized IF matrix IFnorm as:

$$ {\begin{aligned} \text{IFnorm}(i,j) = \frac{\text{IF}(i,j)}{\text{mean}\{\text{IF}(a,b): \lfloor\frac{\text{pos}(b)-\text{pos}(a)}{10,000}\rfloor = \lfloor\frac{\text{pos}(j)-\text{pos}(i)}{1000}\rfloor\}}  \end{aligned}}  $$

where pos(*a*) is the genomic position at the end of RF *a*. Like HiCCUPS, HIFI’s loop-calling algorithm involves two parameters *p* (peak size) and *w* (window size), both measured in bp, with *p*<*w*. For each cell (*i*,*j*) in the HIFI-processed IF matrix at RF resolution, the *peak* around (*i*,*j*) is defined as the submatrix encompassing region [pos(*i*)−*p*..pos(*i*)+*p*]×[pos(*j*)−*p*..pos(*j*)+*p*]. Note that since the size of this submatrix is expressed in bp, the number of cells it contains can vary, depending on the size of the RFs. The average IFnorm value *P*(*i*,*j*) in the peak is then calculated. As with HiCCUPS, *P*(*i*,*j*) is compared to the average IF value found in several types of flanking regions, including (i) *D*(*i*,*j*)—the average IFnorm in the “donut” of size *w* around (*i*,*j*), (ii) *H*(i,j)—the average IFnorm in the left and right flanks of (*i*,*j*), (iii) *V*(*i*,*j*)—the average IFnorm in the top and bottom flanks of (*i*,*j*), and (iv) *BL*(*i*,*j*)—the average score in the region to the bottom left of (*i*,*j*). The score of (*i*,*j*) is then defined as:

$$ \text{score}(i,j) = \frac{P(i,j)}{\text{max}(D(i,j), H(I,j), V(i,j), BL(i,j))}   $$

If score(*i*,*j*)≥minScore, where minScore is a user-defined threshold (we use minScore=1), fragment pair (*i*^′^,*j*^′^)=arg max{IFnorm(*a*,*b*):*a*,*b*∈peak(*i*,*j*)} is identified, and repr(*i*,*j*)=(*i*^′^,*j*^′^) is set as the representative RF pair of the peak. Finally, candidate peaks located within a minimum distance minDist (20 kb used here) of another higher scoring candidate peak are eliminated. The remaining peaks are reported by HIFI.

A false discovery rate (FDR) is assigned to each peak by repeating the peak-finding procedure on a randomly permuted IF matrix (diagonal-wise, i.e., preserving fragment distances). For each distance bin of 10 kb, the observed *score* values are fit to a gamma distribution and used to estimate the FDR of each predicted peak found in the non-permuted data. Similar to HiCCUPS, several pairs of values *p* and *w* were considered to enable the capture of small, sharp peaks as well as broader peaks. Both (*p* = 30 kb, *w* = 60 kb) and (*p* = 50 kb, *w* = 100 kb) were used here. The resulting HIFI chromatin loop calls were combined and sorted based on FDR values.

### Evaluation of chromatin loop-calling approaches

All 3 HindIII-digest GM12878 Hi-C replicates (HIC034, HIC035, and HIC037) from Rao et al. [23] were combined using Juicer [31] (MAPQ value > 30 and Knight-Ruiz matrix balancing [21]) to obtain Hi-C matrices at 10-kb resolution (in.hic format). HiCCUPS was then applied to these matrices using default parameters, yielding a list of 21,940 potential chromatin loops genome-wide.

The top 100 scoring HIFI- and HiCCUPS-predicted chromatin loops (based on FDR values) were identified at each distance bin of 10 kb (Additional file 1: Figure S11). The validity of predicted loops was determined by available CTCF and RNAPII ChIA-PET GM12878 libraries [33]. A predicted loop was considered correct if a proximal ChIA-PET contact was present (allowing up to 10 kb of tolerance for each anchor of the loop).

### Directionality index and TAD boundary prediction

The directionality index (DI) was first described by Dixon et al. [6] to detect directionality bias for interactions across a Hi-C IF matrix. For RF *i*, the DI is usually calculated as follows:

$$ \text{DI}(i)=\text{sign}(B-A) \cdot \left(\frac{(A-E)^{2}}{E}+\frac{(B-E)^{2}}{E}\right),   $$

where $A=\sum _{i-\delta \leq j < i}\text {IF}_{j,i}$, $B=\sum _{i < j \leq i+\delta } \text {IF}_{i,j}$, $E=\frac {(A+B)}{2}$, and *δ* is set to 500 kb. Due to the low coverage at RF resolution Hi-C data, the DI formula yields very noisy predictions. We thus used the following modified version:

$$ \text{DI}^{\prime}=\text{sign}(B-A)\cdot \left(\frac{(A-E)^{2}}{E^{2}}+\frac{(B-E)^{2}}{E^{2}}\right).   $$

This modification transforms terms present in the right parentheses to relative rates and helps to scale the magnitude of the DI. TAD boundaries are defined as RFs whose DI^′^ value is a local maximum or minimum in a window of 21 RFs (51 for MboI analyses) centered around it. In the case of the fixed-binning (*b* = 16) analysis, only RFs at the center of their bin are considered. Due to their low coverage, regions within 2 Mb of a centromere or telomere were excluded. TAD boundaries are then sorted by their absolute DI values and the top 5000 and 25,000 boundaries were selected for HindIII and MboI RF resolutions, respectively.

### Data sources and pre-processing

The following Hi-C datasets were used: from Dixon et al. [6], mESC with HindIII digest (GEO:GSE35156); from Rao et al. [23], GM12878 with HindIII and MboI digest (GEO:GSE63525); and from Bonev et al. [24], mESC with DpnII digest (GEO:GSE96107). For 5C comparisons, the following datasets were used: from Smith et al. [29], GM12878 with HindIII digest (GEO:GSE75634), and from Nora et al. [7], mESC with HindIII digest (GEO:GSE35721). For comparisons to ChIA-PET, the following datasets were used: from Tang et al. [33], CTCF-mediated contacts (GEO:GSM1872886) and RNAPII-mediated contacts (GEO:GSM1872887), and from Fullwood et al. [32], RAD21-mediated contacts (GEO:GSM1436265; replicates averaged). Paired-end tag clusters were binned to hg19 HindIII RFs to ensure comparability with other datasets. Enhancer-promoter (EP) pairs from Thurman et al. [35] were obtained from https://ftp://ftp.ebi.ac.uk/pub/databases/ensembl/encode/integration_data_jan2011/byDataType/openchrom/jan2011/dhs_ gene_connectivity/genomewideCorrs_above0.7_promoterPlusMinus500kb_withGeneNames_32celltypeCategories.bed8.gzhttps://ftp://ftp.ebi.ac.uk/pub/databases/ensembl/encode/integration_data_jan2011/byDataType/openchrom/jan2011/dhs_ gene_connectivity/genomewideCorrs_above0.7_promoterPlusMinus500kb_withGeneNames_32celltypeCategories.bed8.gzhttps://ftp://ftp.ebi.ac.uk/pub/databases/ensembl/encode/integration_data_jan2011/byDataType/openchrom/jan2011/dhs_ gene_connectivity/genomewideCorrs_above0.7_promoterPlusMinus500kb_withGeneNames_32celltypeCategories.bed8.gzhttps://ftp://ftp.ebi.ac.uk/pub/databases/ensembl/encode/integration_data_jan2011/byDataType/openchrom/jan2011/dhs_ gene_connectivity/genomewideCorrs_above0.7_promoterPlusMinus500kb_withGeneNames_32celltypeCategories.bed8.gzhttps://ftp://ftp.ebi.ac.uk/pub/databases/ensembl/encode/integration_data_jan2011/byDataType/openchrom/jan2011/dhs_ gene_connectivity/genomewideCorrs_above0.7_promoterPlusMinus500kb_withGeneNames_32celltypeCategories.bed8.gz. Enhancers and promoters were then binned to their respective RFs. Cohesin-mediated chromatin contacts identified by HiChIP were taken from Supplementary Table 3 of Mumbach et al. [36] and then binned to expected HindIII-digest RFs.

ChIP-seq data from ENCODE [37] and ChromHMM [46] predictions were downloaded from the UCSC Genome browser [56] and binned to HindIII and MboI RFs. For ChromHMM (Fig. 5c, g), only states 1 and 4 were used (to reduce redundancy). CTCF motifs and orientation were identified in a similar manner to Fundenberg et al. [57] using HOMER [58] and the “CTCF_known1” PWM [59]. CTCF peaks, identified by ChIP-seq, were assigned forward or reverse strand orientations based on the orientation of overlapping CTCF motifs. If both orientations were found to reside within a peak, then one orientation would be randomly chosen. Peaks with no overlapping CTCF motif were discarded.

## Supplementary information

**Additional file 1** Supplementary information.

**Additional file 2** Review history.
