# Supplementary material for: HIFI: estimating DNA-DNA interaction frequency from Hi-C data at restriction-fragment resolution
Source: Genome Biol. 2020 Jan 14;21:11. doi: 10.1186/s13059-019-1913-y (PMC6961295; doi:10.1186/s13059-019-1913-y)
Supplement: Supplementary file 1 — Additional file 1 Supplementary information. [file 13059_2019_1913_MOESM1_ESM.pdf]

# Supplementary Figures

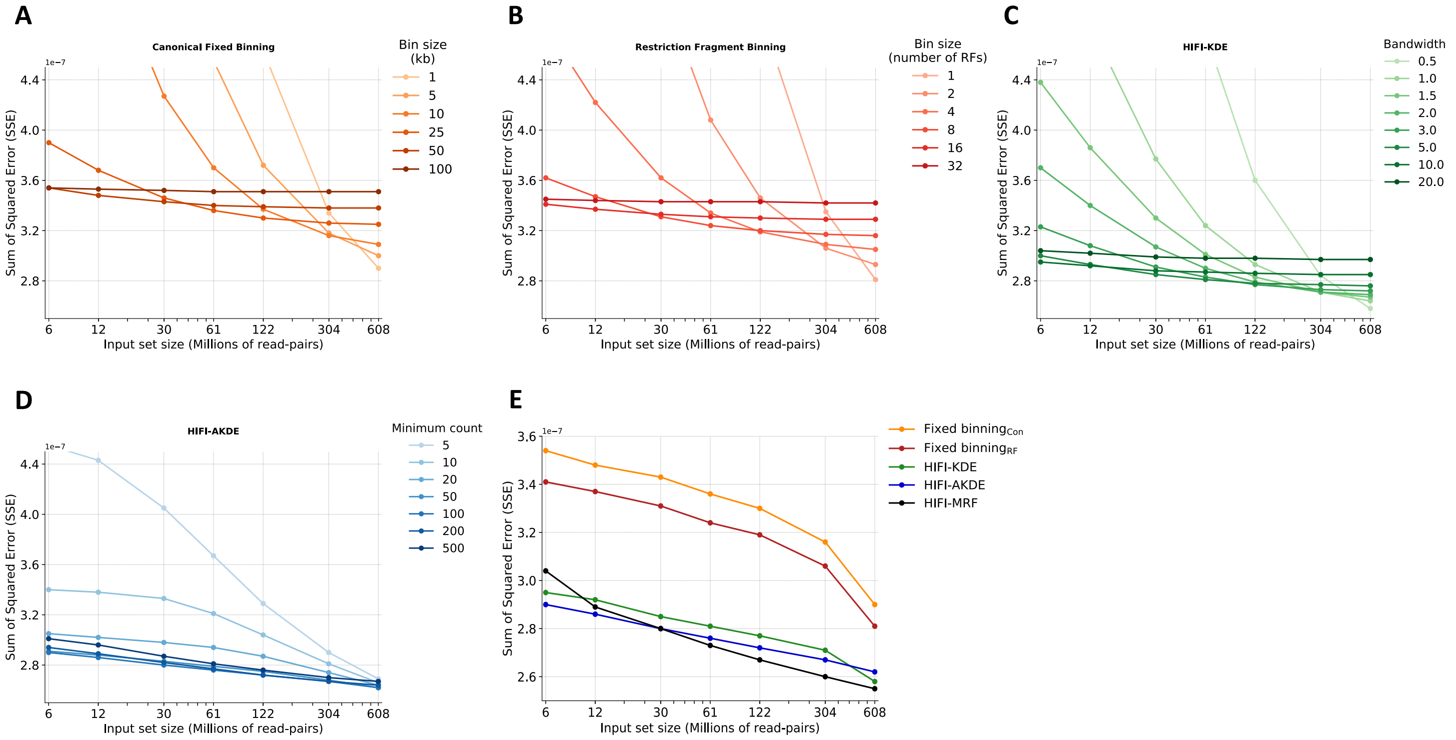


**Fig. S1 Cross-validation of fixed-binning and HIFI methodologies.**
**A**) [Reproduced from Fig. 1B, to facilitate comparison.] Cross-validation error for canonical fixed-binning approaches, for different bin sizes, as a function of coverage. **B**) Same analysis, performed on fixed-binning algorithm where bin size is expressed in terms of the number of restriction fragments rather than in kb. **C**) Cross-validation error for HIFI-KDE, at different bandwidth values. **D**) Cross-validation error for HIFI-AKDE, at different Minimum Count values. **E**) Comparison of most accurate parameter sets at various input set sizes for all inference methodologies (based on SSE). We observe that HIFI-MRF outperforms all other approaches described for most input set sizes.

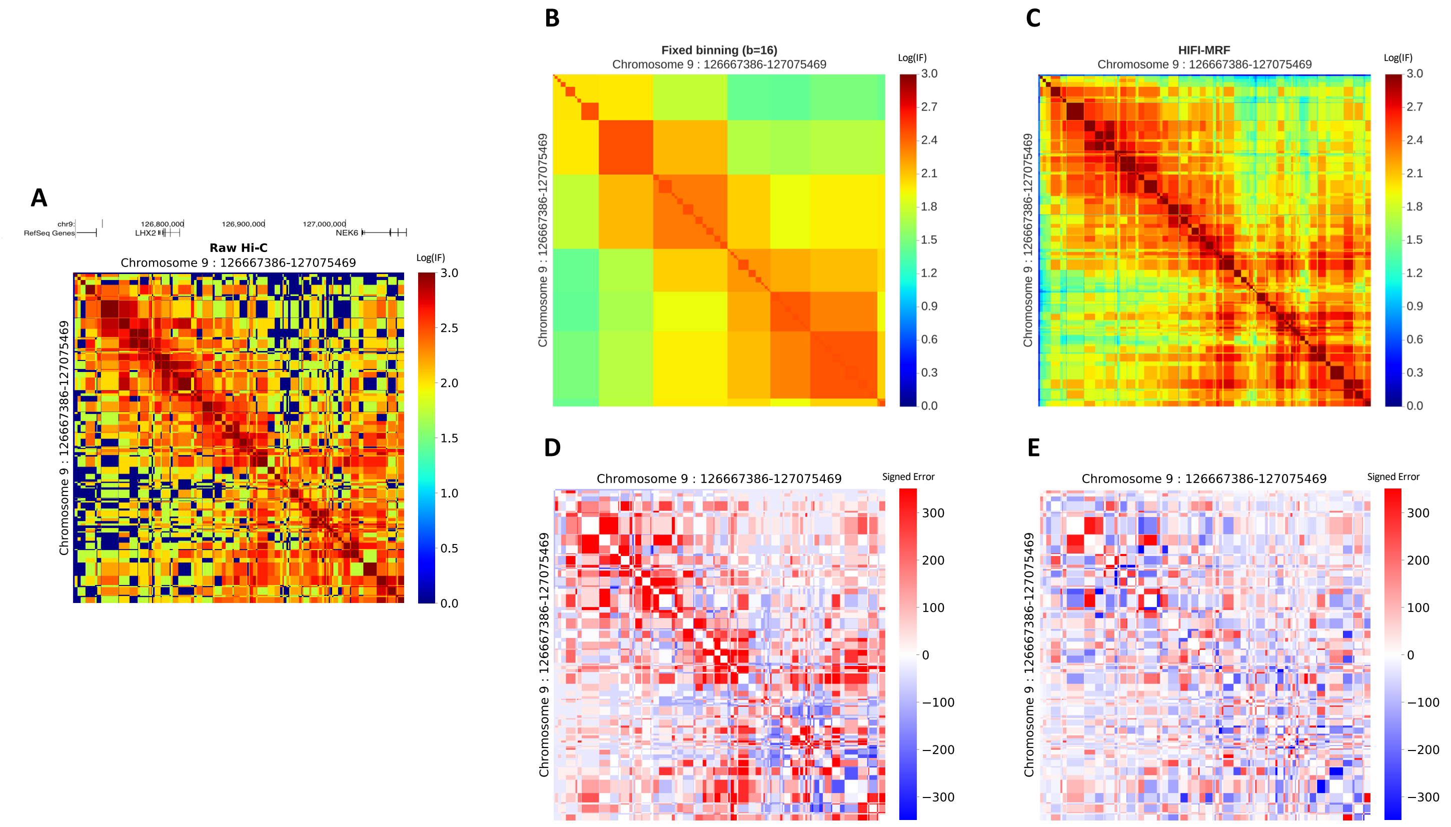


**Fig. S2 Fixed-binning approach vs. HIFI-MRF.**
**A**) Raw Hi-C IF matrix for the NEK6 locus at HindIII RF-resolution. Inferred Hi-C IF matrices resulting from fixed-binning (b=16 RFs) and HIFI-MRF approaches is shown in (**B**) and (**C**), respectively. Signed error matrices (resulting from the subtraction of the raw Hi-C matrix [**A**] by either [**B**] or [**C**]) are shown for fixed binning (**D**) and HIFI-MRF (**E**). A noticeable reduction in error is observed for the HIFI-MRF signed error (**E**) when compared to the fixed-binning (**D**).

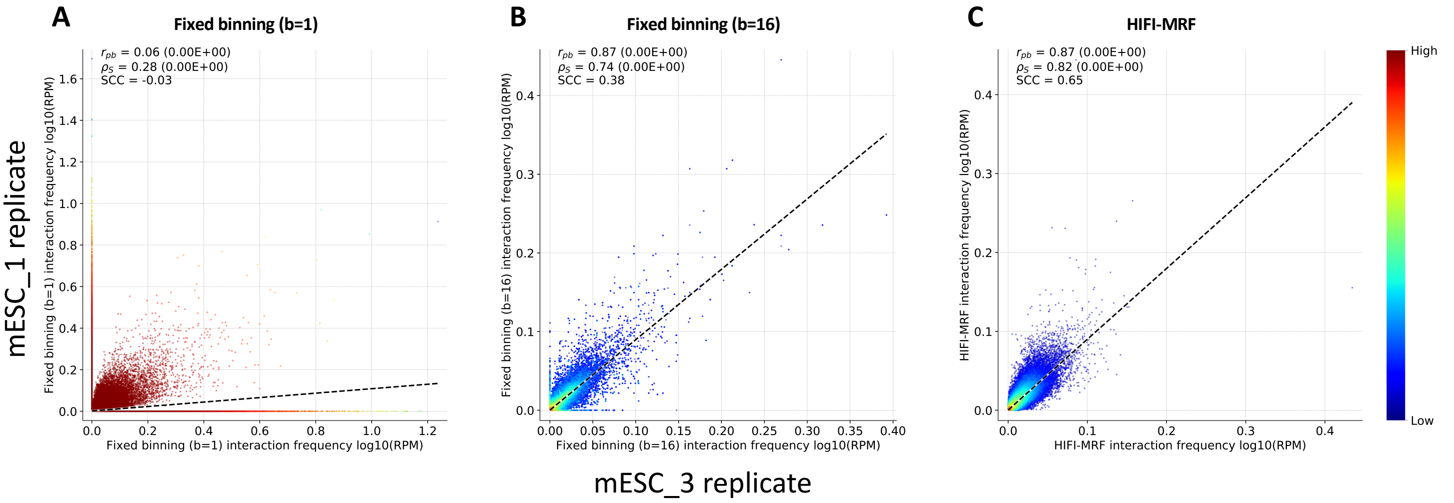


**Fig. S3 mESC-DpnII replicate analysis of fixed-binning approach vs. HIFI-MRF.**
Comparison of the first and third mESC Hi-C replicates (mESC_1 and mESC_3, respectively) from Bonev et al. (2017) [1]. Five million restriction-fragment pairs within a genomic distance of 1 Mb were randomly sampled from the expected DpnII-digest of mESC chr14. Compared to fixed-binning approaches of 1 (**A**) or 16 (**B**) fragments, HIFI-MRF (**C**) is shown to provide the highest replicate similarity (based on the Pearson $r_{pb}$, Spearman $\rho_s$, and SCC correlations between mESC_1 and mESC_3).

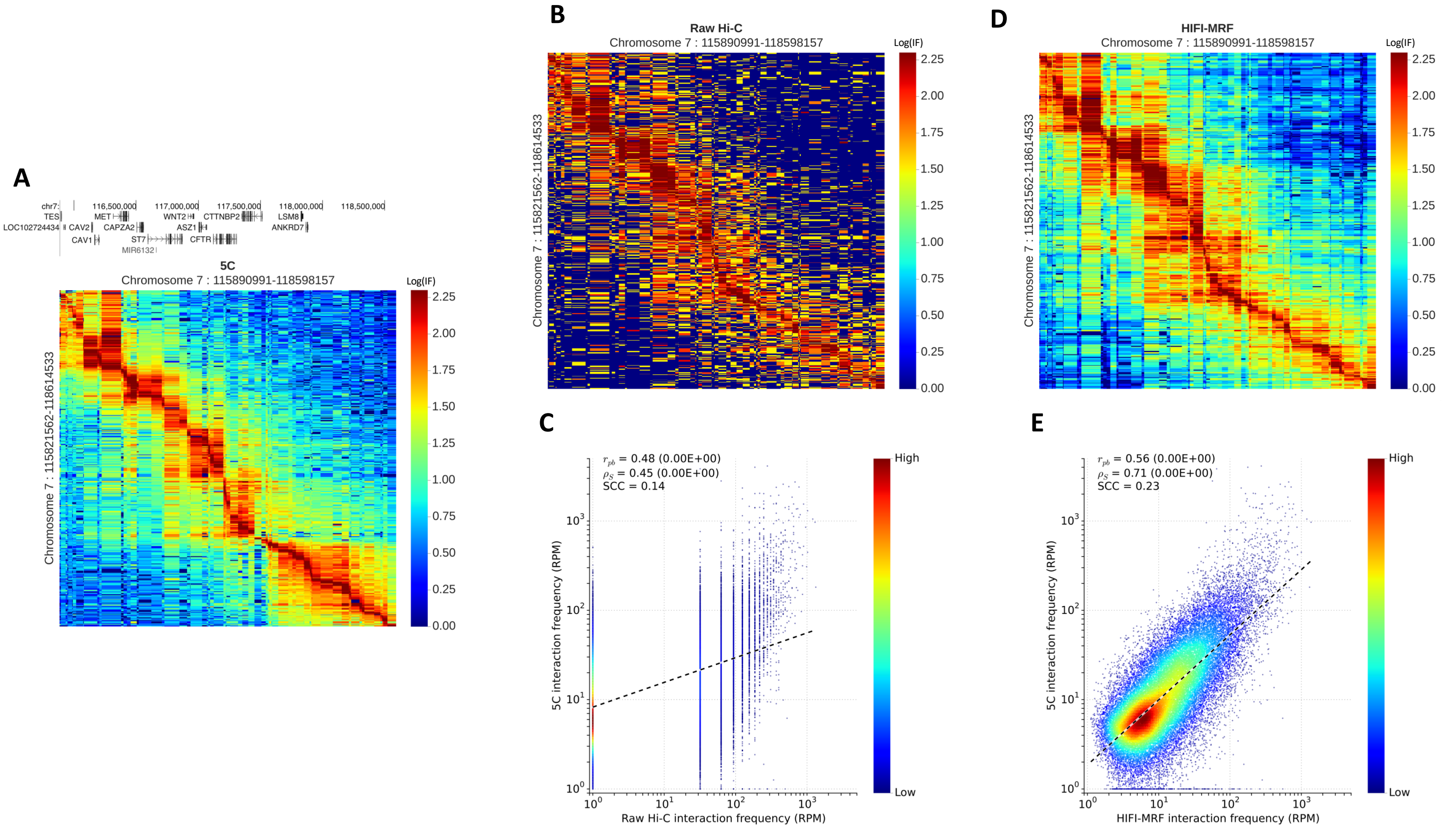


**Fig. S4 Recapitulation of 5C observations by HIFI-MRF (GM12878-chr7 example).**
5C data for chr7:115890991-118598157 in GM12878 cells (**A** [2]). (**B**) Raw HiC data [3] for the corresponding region. (**C**) Correlation of raw HiC data with 5C data for this region (Pearson $r_{pb} = 0.48$, p-value$< 10^{-16}$; Spearman $\rho_s = 0.45$, p-value$< 10^{-16}$); SCC = 0.14). (**D**) HIFI-MRF inferred IFs for the same region. (**E**) Correlation of HIFI-MRF inferred IFs with 5C data for this region (Pearson $r_{pb} = 0.56$, p-value$< 10^{-16}$; Spearman $\rho_s = 0.71$, p-value$< 10^{-16}$); SCC = 0.23). Also observe how HIFI-MRF processed data displays TADs and a decay constant profile similar to those observed by 5C.

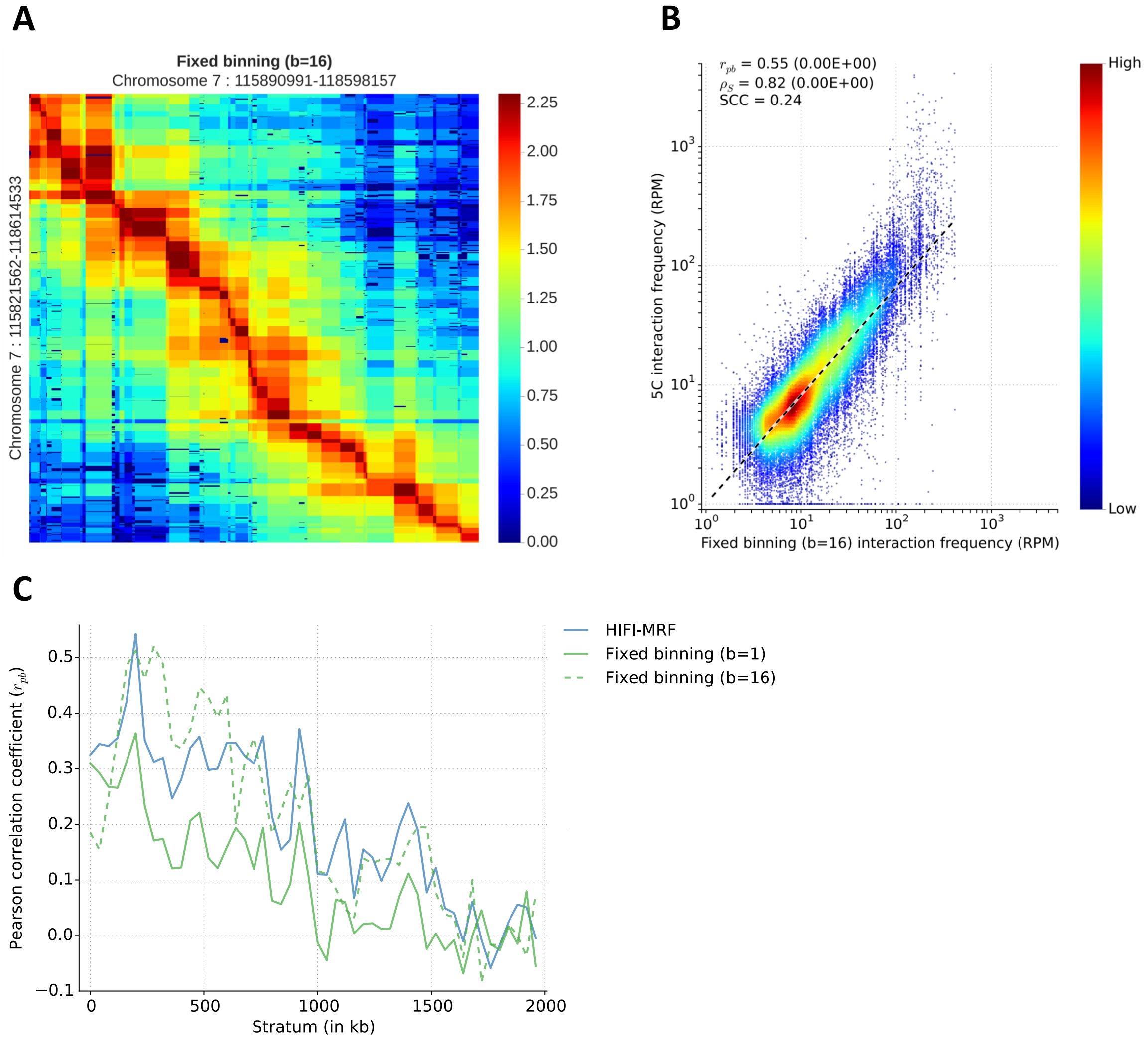


**Fig. S5 Fixed-binning recapitulation of 5C contacts (GM12878-chr7 example).**
(**A**) Fixed binning (b = 16) analysis of Hi-C data for GM12878-chr7 RF-pairs [2].
(**B**) Comparison of observed 5C and fixed-binning (b = 16) Hi-C values for the region (Pearson $r_{pb} = 0.55$, p-value$< 10^{-16}$; Spearman $\rho_s = 0.82$, p-value$< 10^{-16}$); SCC = 0.24).
(**C**) Distribution of Spearman $\rho_s$ values by genomic distance for Hi-C contacts resulting from HIFI-MRF and fixed binning. Both HIFI-MRF and fixed-binning perform similarly for this example.

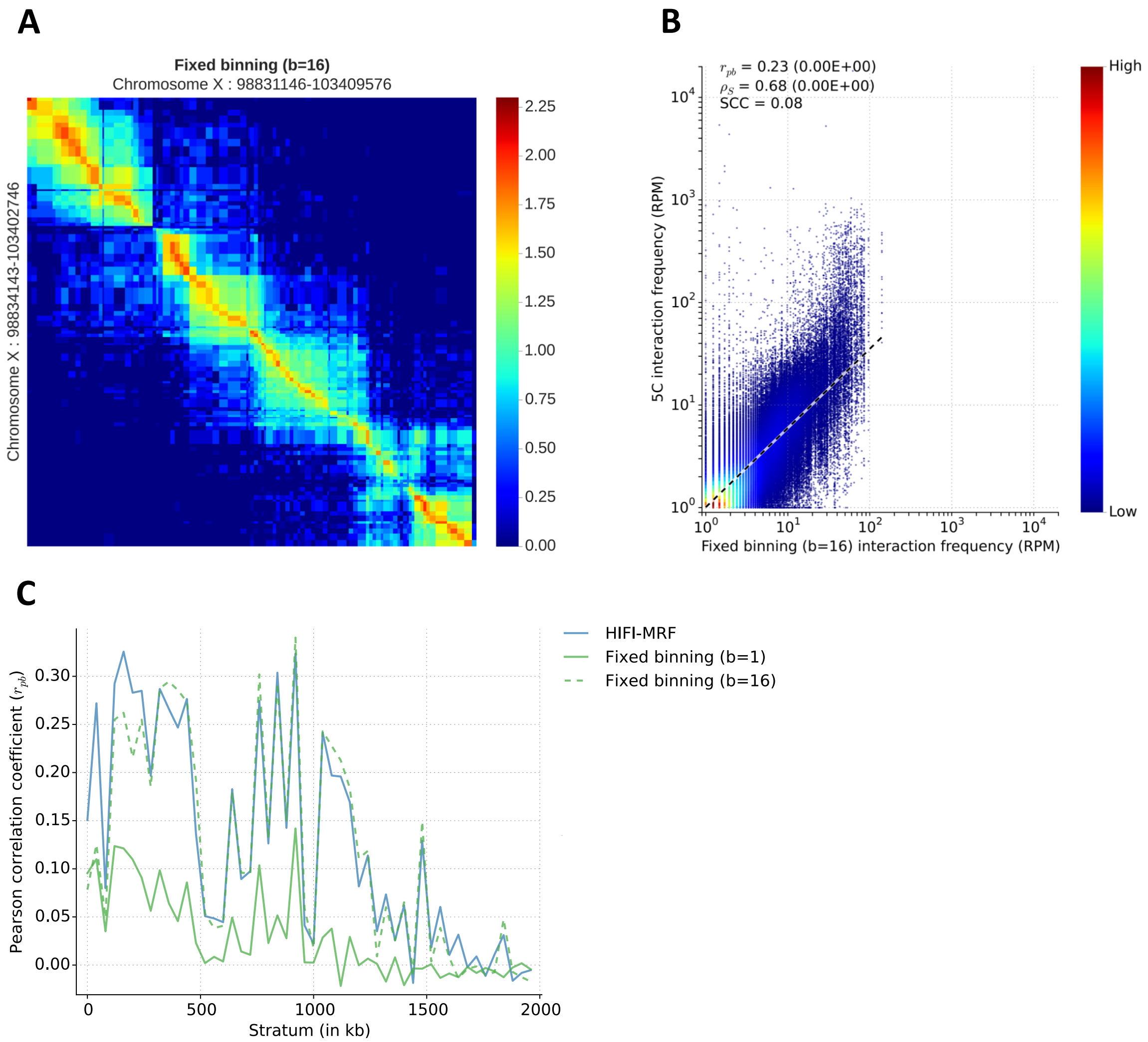


**Fig. S6 Fixed-binning recapitulation of 5C contacts (mESC Xist locus).**
**A**) Fixed binning (b = 16) analysis of Hi-C data for a 4.5 Mb region surrounding Xist in mESC.
**B**) Comparison of observed 5C [4] and fixed-binning (b = 16) Hi-C values for the region (Pearson $r_{pb} = 0.23$, p-value$< 10^{-16}$; Spearman $\rho_s = 0.68$, p-value$< 10^{-16}$); SCC = 0.08).
**C**) Distribution of Spearman $\rho_s$ values by genomic distance for Hi-C contacts resulting from HIFI-MRF and fixed binning. Overall, due to the low SCC [5] value and similarity in distributions of panel **C**, most of the improvement by either method is driven by distance dependencies between contacts.

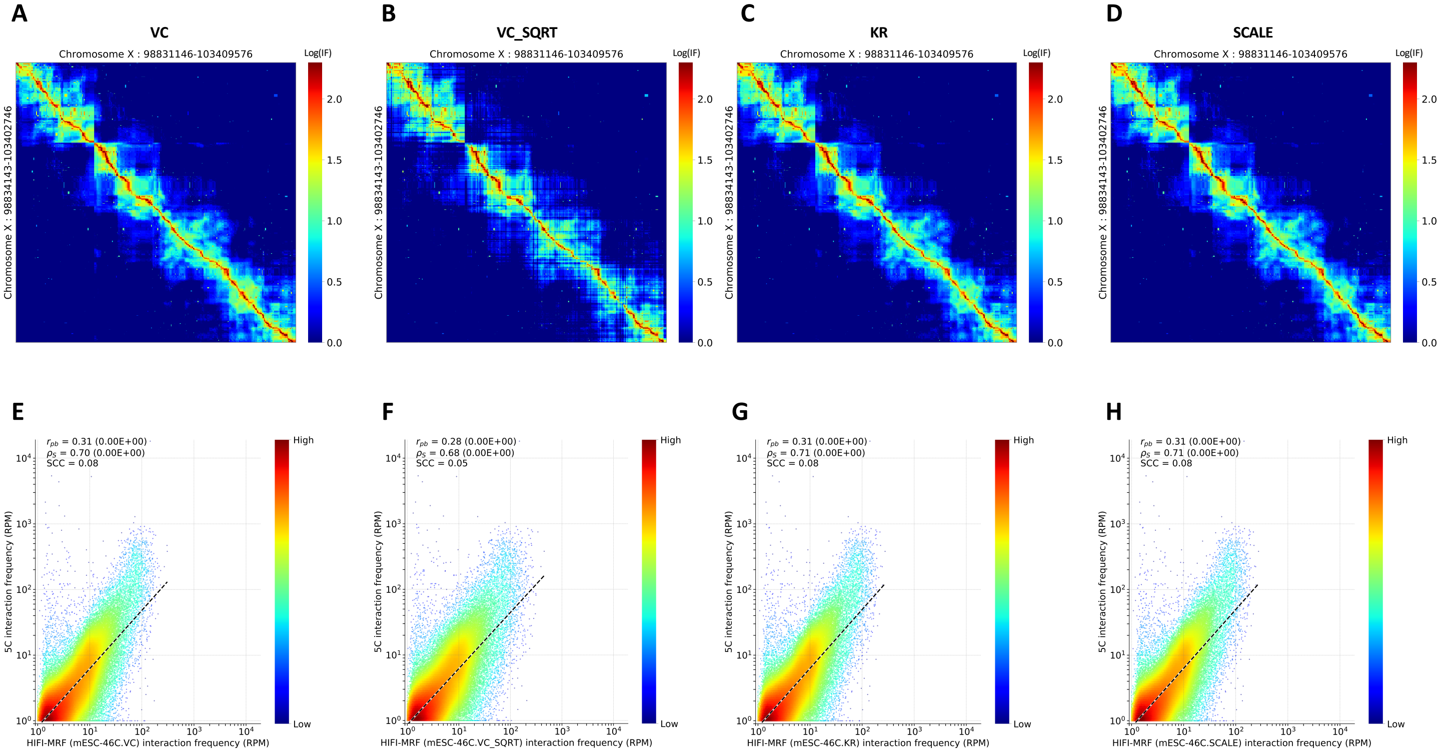


**Fig. S7 Fragment-bias normalization comparison of the mESC Xist locus.**
Raw HIFI-MRF estimates for the 4.5 Mb region surrounding Xist in mESC were provided to Juicer (v1.11.09) [6] in the 'short with score' format. Juicer implementations of the Vanilla Coverage (VC — **A**), SQuare RooT of Vanilla Coverage (VC_SQRT — **B**), Knight and Ruiz (KR — **C**) matrix balancing [7], and SCALE (**D**) fragment-bias normalization algorithms result in similar heatmaps. For all heatmaps, both TADs and the decay constant profile of target 5C values (Fig. 2A) are recapitulated. The *bottom row* consists of correlations between target 5C values against HIFI-MRF estimates normalized by either the VC (**E**), VC_SQRT (**F**), KR (**G**), or SCALE (**H**) algorithms. Overall, no significant difference is observed in the accuracy of HIFI-estimates (based on the Pearson $r_{pb}$, Spearman $\rho_s$, and SCC correlations between 5C and HIFI-MRF IFs) across the various normalization algorithms.

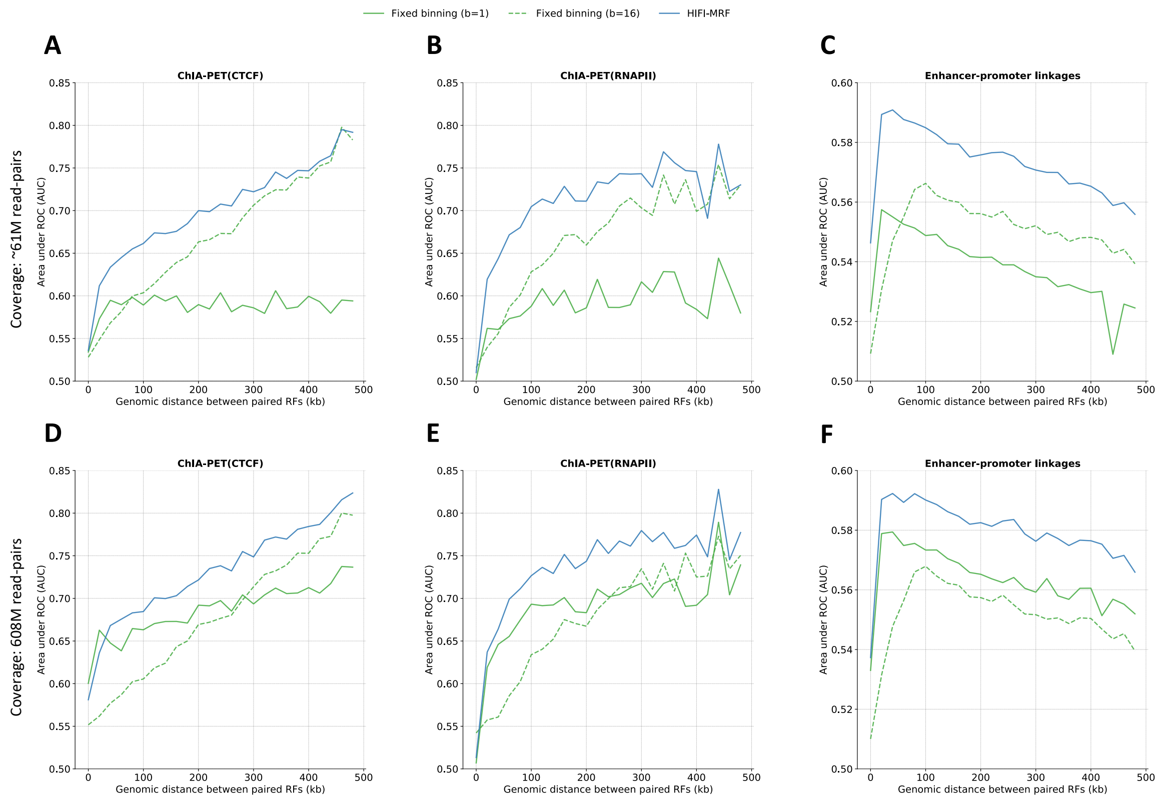


**Fig. S8 Positive/negative RF contact delineation analysis (genome-wide).**
Repetition of the analysis shown in Fig. 4, but this time for the whole genome. See caption of Fig. 4.

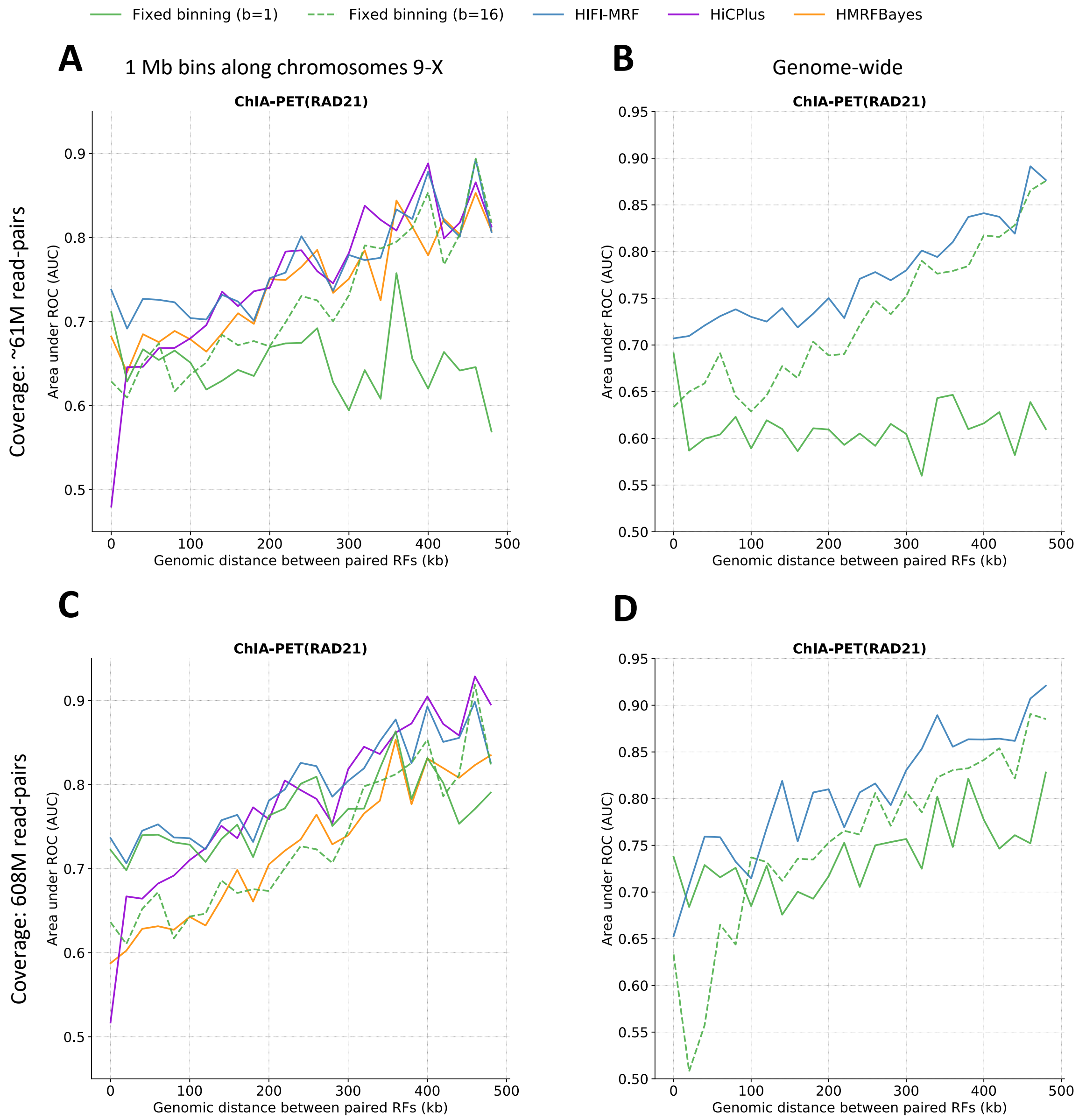


**Fig. S9 Positive/negative RF contact delineation analysis (RAD21).**
Repetition of the analysis presented in Fig. 4, this time for ChIA-PET RAD21 data [8]. HIFI-MRF is found to provide more accurate (based on AUROC) predictions of RF-pair classification (positive vs. negative) compared to other inference methods for contacts located less than 100 kb apart, but is on par with HiCPlus and HMRFBayes at larger distances.

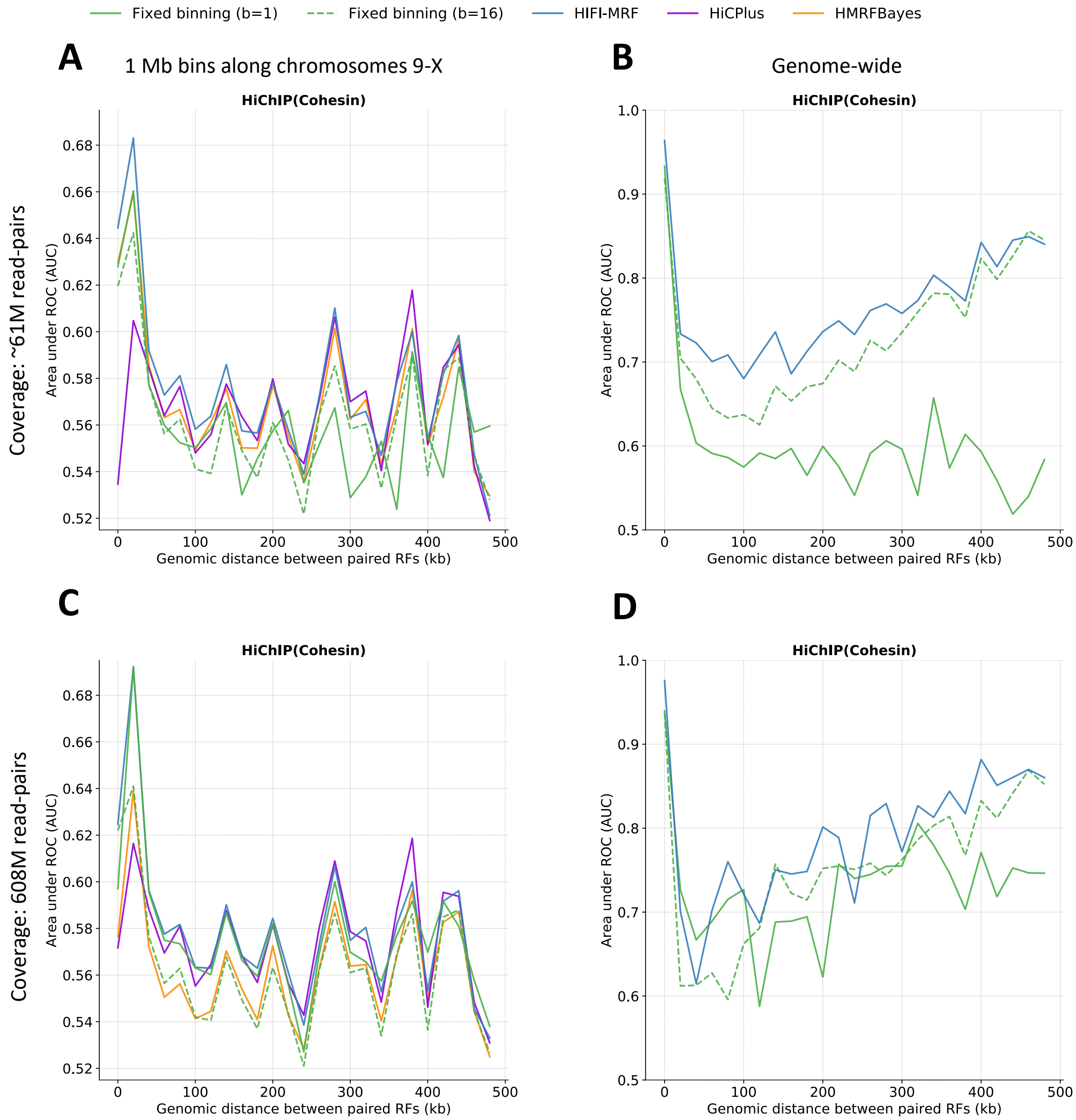


**Fig. S10 Positive/negative RF contact delineation analysis (HiChIP).**
Repetition of the analysis presented in Fig. 4, this time for HiChIP cohesin data [9]. HIFI-MRF is found to be on par or provide more accurate (based on AUROC) predictions of RF-pair classification (positive vs. negative) compared to other inference methods.

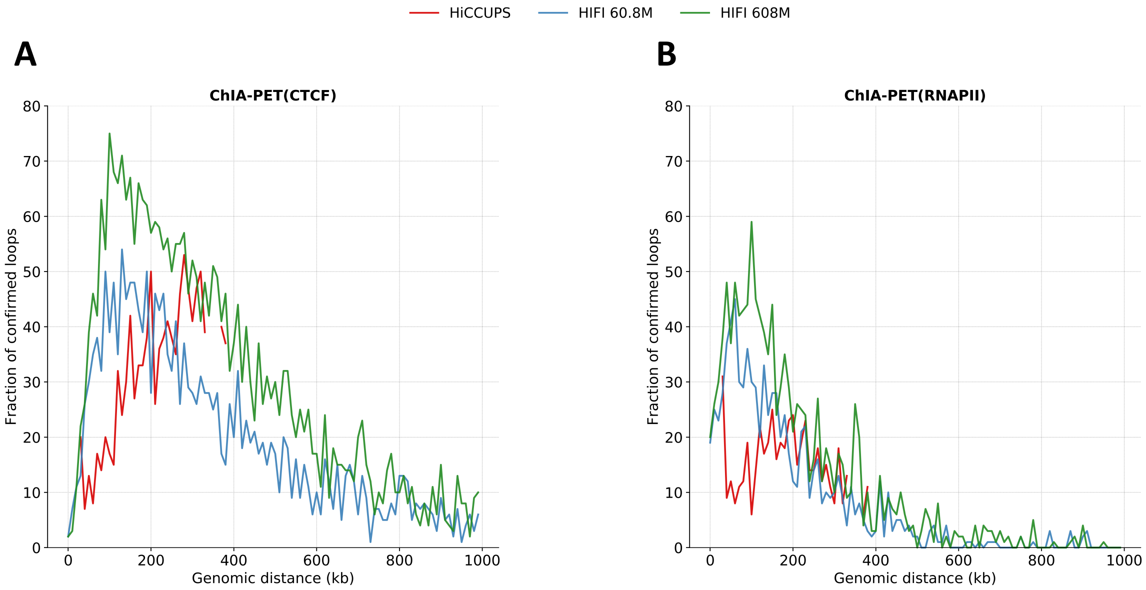


**Fig. S11 Comparison of predicted chromatin loops by HiCCUPS and HIFI.**
For each distance bin of 10 kb, the predicted loops are confirmed by CTCF (**A**) and RNAPII (**B**) ChIA-PET data sets, allowing a tolerance of 10 kb. HiCCUPS was applied to non-smoothed, normalized Hi-C data. HIFI implements a similar loop-calling program to HiCCUPS that is applied directly to HIFI-processed Hi-C data (60.8M and 608M read-pair coverage). Note — missing data represents 10 kb distance bins where HiCCUPS did not provide a sufficient number (100) of predicted chromatin loops.

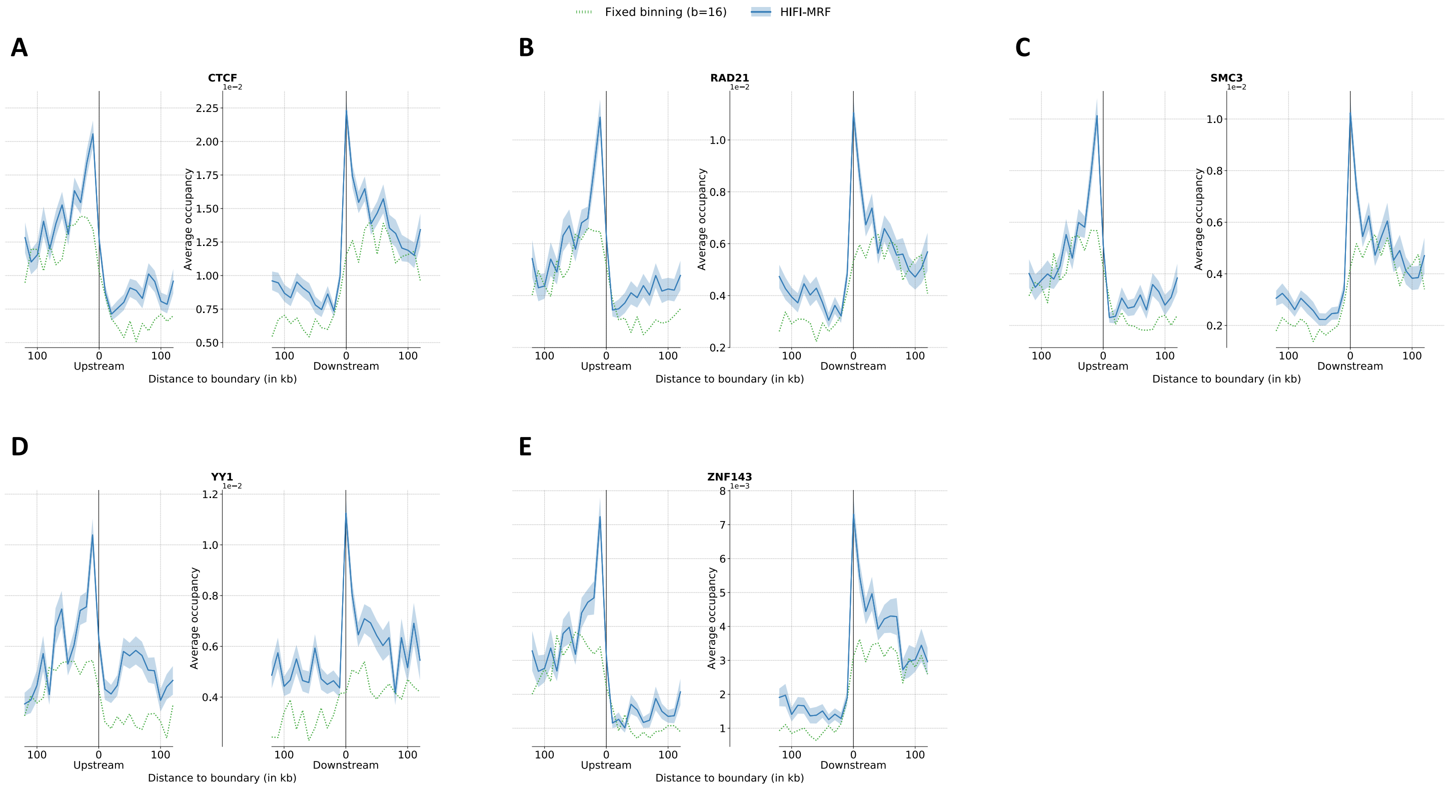


**Fig. S12 GM12878-HindIII RF-resolution TAD boundary occupancy by architectural proteins** Architectural proteins CTCF (**A**) , RAD21 (**B**), SMC3 (**C**), YY1 (**D**), and ZNF143 (**E**) are strongly enriched near TAD boundaries, followed by a significant depletion in occupancy within TADs.

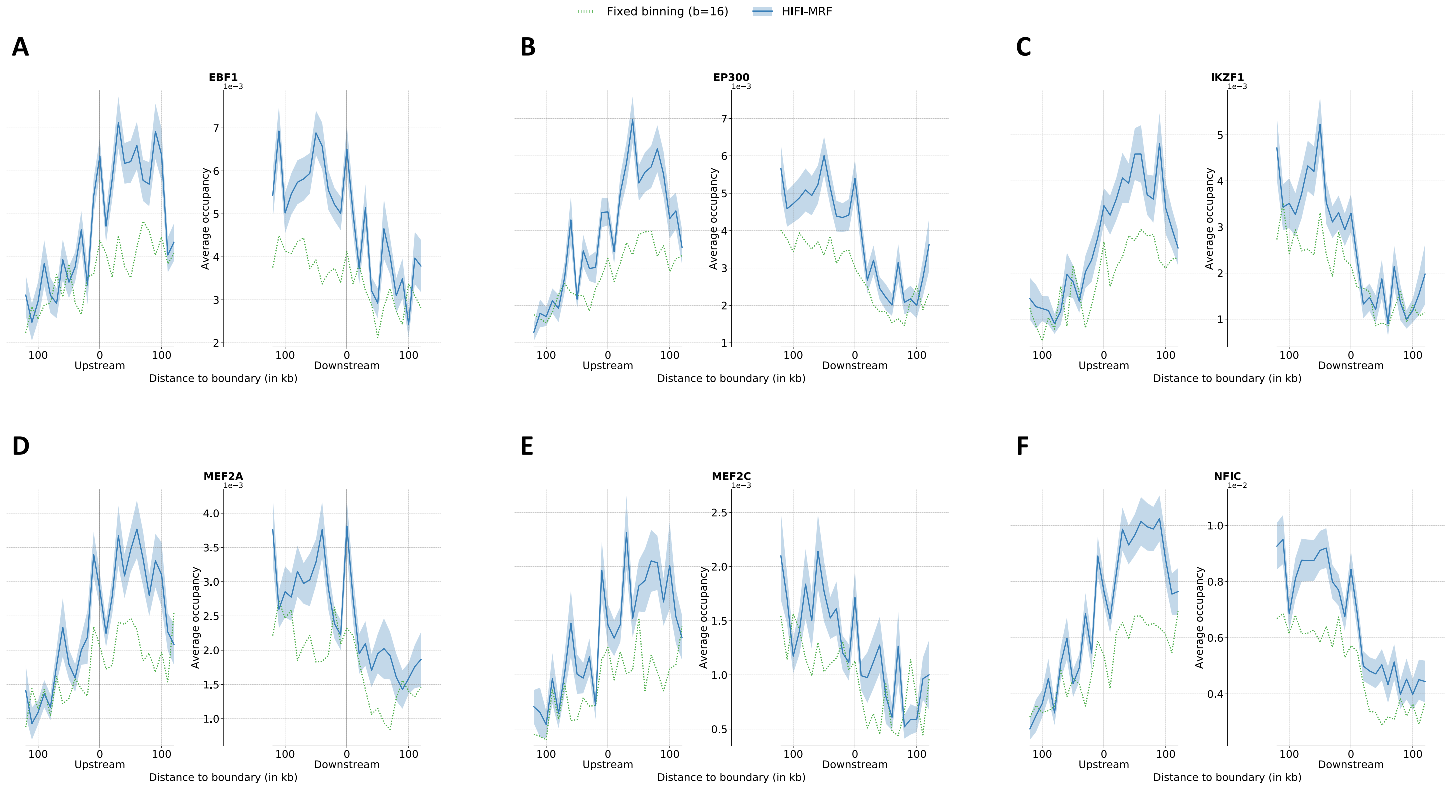


**Fig. S13 GM12878-HindIII RF-resolution within-TAD enrichment by selected transcription factors.** Transcription factors EBF1 (**A**), EP300 (**B**), IKZF1 (**C**), MEF2A (**D**), MEF2C (**E**), and NFIC (**F**) show an enrichment within TADs, compared to outside of TAD boundaries.

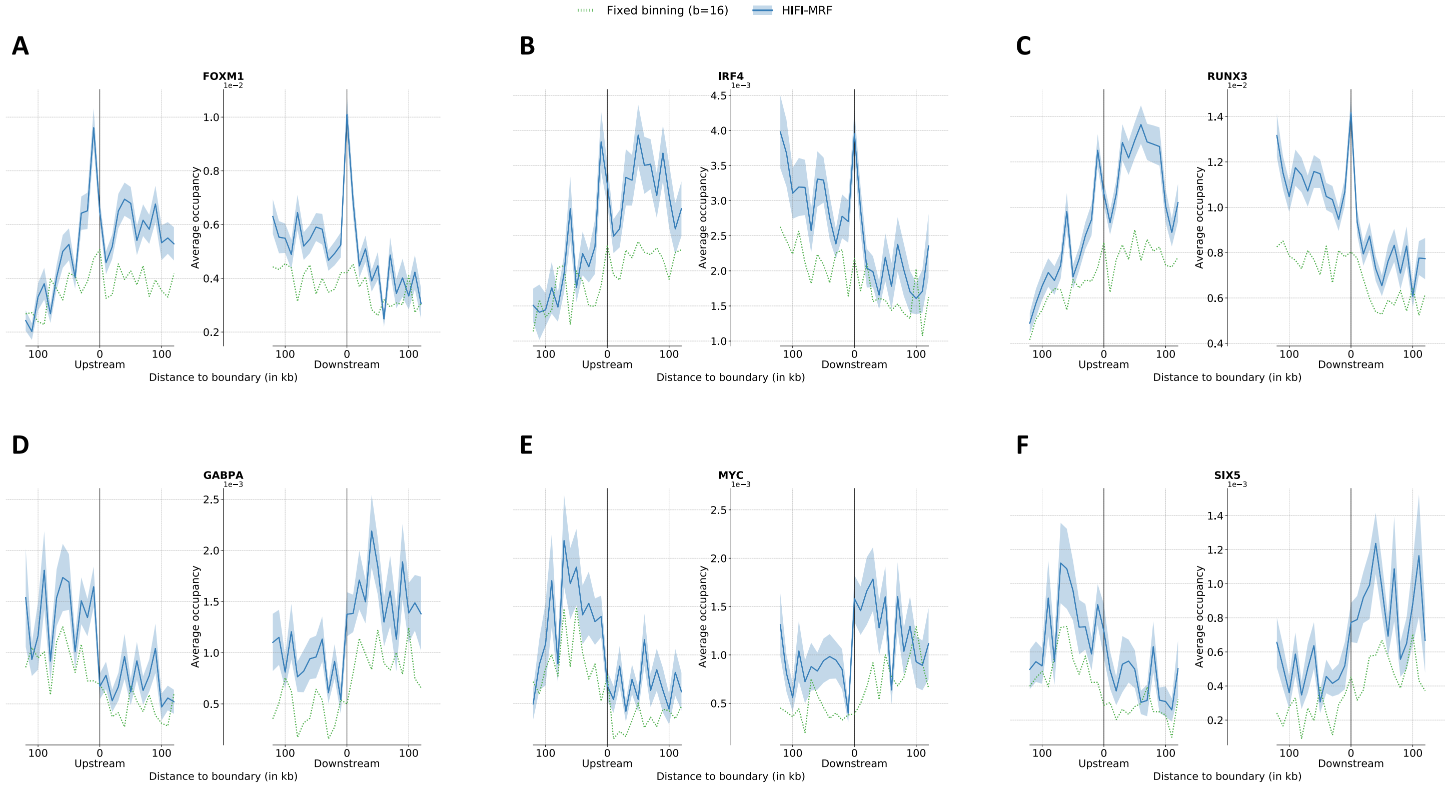


**Fig. S14 GM12878-HindIII RF-resolution TAD boundary occupancy of selected transcription factors.** Transcription factors FOXM1 (**A**), IRF4 (**B**), and RUNX3 (**C**) exhibit a strong enrichment specific to TAD boundaries, in addition to an enrichment within TADs. On the opposite, transcription factors GABPA (**D**), MYC (**E**), and SIX5 (**F**) show a minor depletion within TADs compared to outside of TADs.

# References


[1] Bonev, B., Mendelson Cohen, N., Szabo, Q., Fritsch, L., Papadopoulos, G.L., Lubling, Y., Xu, X., Lv, X., Hugnot, J.P., Tanay, A., Cavalli, G.: Multiscale 3D Genome Rewiring during Mouse Neural Development. Cell **171**(3), 557–57224 (2017)

[2] Smith, E.M., Lajoie, B.R., Jain, G., Dekker, J.: Invariant TAD Boundaries Constrain Cell-Type-Specific Looping Interactions between Promoters and Distal Elements around the CFTR Locus. Am J Hum Genet **98**(1), 185–201 (2016)

[3] Rao, S.S., Huntley, M.H., Durand, N.C., Stamenova, E.K., Bochkov, I.D., Robinson, J.T., Sanborn, A.L., Machol, I., Omer, A.D., Lander, E.S., Aiden, E.L.: A 3D map of the human genome at kilobase resolution reveals principles of chromatin looping. Cell **159**(7), 1665–1680 (2013)

[4] Nora, E.P., Lajoie, B.R., Schulz, E.G., Giorgetti, L., Okamoto, I., Servant, N., Piolot, T., van Berkum, N.L., Meisig, J., Sedat, J., Gribnau, J., Barillot, E., Blüthgen, N., Dekker, J., Heard, E.: Spatial partitioning of the regulatory landscape of the X-inactivation centre. Nature **485**(7398), 381–385 (2012)

[5] Yang, T., Zhang, F., Yardımcı, G.G., Song, F., Hardison, R.C., Noble, W.S., Yue, F., Li, Q.: HiCRep: assessing the reproducibility of Hi-C data using a stratum-adjusted correlation coefficient. Genome Res **27**(11), 1939–1949 (2017)

[6] Durand, N.C., Shamim, M.S., Machol, I., Rao, S.S., Huntley, M.H., Lander, E.S., Aiden, E.L.: Juicer Provides a One-Click System for Analyzing Loop-Resolution Hi-C Experiments. Cell Syst **3**(1), 95–98 (2016)

[7] Knight, P., Ruiz, D.: A fast algorithm for matrix balancing. IMA J Numer Anal **33**, 1029–1047 (2013)

[8] Heidari, N., Phanstiel, D.H., He, C., Grubert, F., Jahanbani, F., Kasowski, M., Zhang, M.Q., Snyder, M.P.: Genome-wide map of regulatory interactions in the human genome. Genome Res **24**(12), 1905–1917 (2014)

[9] Mumbach, M.R., Rubin, A.J., Flynn, R.A., Dai, C., Khavari, P.A., Greenleaf, W.J., Chang, H.Y.: HiChIP: Efficient and sensitive analysis of protein-directed genome architecture. Nat Methods **13**(11), 919–922 (2017)
