# Supplementary material for: HIFI: estimating DNA-DNA interaction frequency from Hi-C data at restriction-fragment resolution
Source: Genome Biol. 2020 Jan 14;21:11. doi: 10.1186/s13059-019-1913-y (PMC6961295; doi:10.1186/s13059-019-1913-y)
Supplement: Supplementary file 2 — Additional file 2 Review history. [file 13059_2019_1913_MOESM2_ESM.docx]

Review History

**First round of review**

**Reviewer 1**

**Were you able to assess all statistics in the manuscript, including the appropriateness of statistical tests used?**

Yes: The statistical tests used in this study are appropriate.

**Were you able to directly test the methods?**

Yes

**Comments to author:**

In this paper, the authors described a new method HIFI to estimate chromatin interaction from Hi-C data at fragment resolution. This work addresses an important research question. The proposed methodology is interesting. The authors performed comprehensive analysis to benchmark the performance of HIFI with existing methods. The paper is also well written. However, I still have a few comments and suggestions which may further improve the current manuscript. Here are my specific comments.

Major comments:

1. The major goal of this work is to perform Hi-C data analysis at fragment resolution. However, the authors only provided new estimate of fragment resolution interaction frequency. I feel this work is not complete. Without well-designed methods for fragment resolution Hi-C data normalization and significant chromatin interaction detection, it is difficult to interpret the output of HIFI. The potential impact of HIFI is limited. It would be a key contribution if the authors can provide a complete pipeline for Hi-C data analysis at fragment resolution for both 4 bp cutter and 6 bp cutter. In addition, for HindIII Hi-C data, Jin et al, Nature, 2013 study (PMID: 24141950) also did fragment resolution analysis. It is necessary to carefully compare HIFI with Jin et al's method.

2. Most results in this paper are based on Rao et al GM12878 data with 6bp cutter HindIII. However, the major deliverable in the Rao et al 2014 study is the in situ Hi-C experiment protocol and GM12878 data with 4 bp cutter MboI. GEO GSE6352 lists two replicates of GM12878 in situ Hi-C data (primary and replicate). The authors can use one as the training set, and the other as the testing set. More importantly, almost all recent Hi-C studies use in situ Hi-C with 4 bp cutter MboI or DpnII. Since the goal of this work is to estimate fragment resolution Hi-C contact frequency, I hope the author can present in situ Hi-C with 4 bp cutter in the main text, and move all results based on 6 bp cutter into supplementary.

3. In the section "validation again 5C data", the authors can use mESC Hi-C data from Bonev et al Cell 2017 paper (PMID: 29053968) and GM12878 in situ Hi-C data from Rao et al 2014. Those data are the deepest Hi-C data to date and with better quality than the Hi-C data currently used in this study.

4. In addition to comparison with ChIA-PET data, I hope the authors can also compare HIFI-predicted Hi-C contact frequency with GM12878 HiChIP data (PMID: 28945252), which is the current state-of-the-art measurement of enhancer-promoter interactions.

5. Page 9, line 52. The authors used a matrix-balancing algorithm to do fragment resolution bias normalization, which can be problematic. In literature, matrix-balancing algorithm has been applied to equally-binned Hi-C data, but not for fragment resolution Hi-C data. In addition, a recent study (Gilgenast and Phillips-Cremins, Cell Systems, 2019, PMID: 30904376) has shown that the equal visibility assumption does not apply to fragment resolution 5C data. So I would suspect that matrix-balancing algorithm is not appropriate for fragment resolution Hi-C data either. I hope the authors either develop a new normalization method, or use established methods in two previous studies (Yaffe and Tanay, Nature Genetics, 2011. PMID: 22001755 and Jin et al, Nature, 2013. PMID: 24141950)

6. A key feature from high resolution Hi-C data is chromatin loop identified by HiCCUPS. It would be more convincing if HIFI-predicted chromatin interactions can help to improve statistical power of chromatin loop detection.

7. I don't fully understand the method used in Figure 4. HIFI is used to estimate fragment resolution Hi-C contact frequency. Similar to HIFI, HiCPlus can be used to enhancer Hi-C data resolution. Different from HIFI and HiCPlus, HMRFBayes is a significant interaction caller. How did the authors obtain significant chromatin interactions from HIFI-predicted and HiCPlus-enhanced Hi-C data?

Minor comments:

1. Page 3, line 61 - page 4, line 6. The authors mentioned that Hi-C data with billion reads still have very low average reads between each fragment end pairs. It would be more informative to show the average reads per fragment end pairs for each 1D genomic distance, and the percentage of fragment end pairs without any read for each 1D genomic distance. At fragment resolution, read count for pairs >1Mb or between different TADs is very sparse, therefore genome-wide average is not very informative.

2. Page 3, line 40 - 44. I don't quite understand the approach 1. For fixed-binning approach, when combining consecutive fragments into bins, the unit of analysis is bin, not fragment. So one will only use the summation of all reads between bin pairs, without taking average for fragment pairs.

3. Page 5, line 8. Typo: "HiPlus" should be "HiCPlus".

4. Page 5, line 8 - line 11. The authors need to present the HiCPlus results. The current wording for the comparison with HiCPlus is very vague. It would be more convincing if the authors show concrete numerical results for HiCPlus.

5. Page 8, line 55. It takes around one hour per chromosome at HindIII resolution. What is the computational cost for analysis at MboI fragment resolution? Is it practical to apply HIFI to most in situ Hi-C data with 4 bp cutter?

6. Page 10, line 25. How did the author choose the bandwidth parameter h?

7. Page 10, line 44. "MinimumCount" is a user-defined parameter. I hope the authors can provide some justification/guidance on how to choose this parameter in practice.

8. HIFI-MRF relies on some optimization algorithms. What is the reproducibility of HIFI-MRF output? Is it robust to initial values and random seeds?

**Reviewer 2**

**Were you able to assess all statistics in the manuscript, including the appropriateness of statistical tests used?**

Yes: OK

**Were you able to directly test the methods?**

No

**Comments to author:**

Cameron et al. proposed a HIFI program for Hi-C data analysis. The idea is to impute deep Hi-C data at fragment resolution from sparse data. Conceptually it is possible to do this if data from neighboring fragments are taken. The paper compared several approaches and landed on the Markov random fields (MRF) method with the best outcome. The direct validation was done by comparing to two 5C data. Indirect validation was done by comparing to the ChIA-PET (CTCF and PolII) and E-P link (based on DHS correlation). The author also showed some examples using the enhanced data to analyze the features near TAD or sub-TAD boundaries.

Overall this is an interesting approach and good addition to the Hi-C analysis field. It is potentially useful when only low-depth Hi-C data is available. The computational method seems to be largely reasonable. On the other hand, from the point of view of users, this paper will benefit from providing more benchmarks and comparative analyses. A little restructuring of the results can be helpful to readers, too. Below are my comments.

1. The authors listed 4 HIFI method but starting from Fig. 2, Fixed bin approaches are quite obvious. HIFI-KDE or HIFI-AKDE are almost never mentioned after Fig. 1. The authors clearly prefer HIFI-MRF. I suggest the authors to move some results to supp and keep the readers focusing on HIFI-MRF.

2. Relate to Fig. 1, choice of test data. For cross validation, the authors reserve 20% data as test set and downsampled the rest data at different level and computed the correlation between HIFI-predicted data to the test data. I feel a little confused why only choosing 20% as test data. For example, the HIFI predicted data from 80% data will be cross-validated to un-enhanced low depth data, which can be misleading.

3. Reproducibility. Published deep Hi-C data are usually from multiple biological replicates. Can the authors assess how reproducible their result is from different biological replicates? E.g. showing the results on the two 5C locus.

4. Lower limit of method. The depth of data obviously cannot be too low. As a potential user, I will be interested in knowing about the limitations of the method. What is the low limit of sequencing depth? Results from down-sampled data on the 5C results will be helpful.

5. Relate to Fig. 4, loop calling method? I did not find the detailed method on how the authors call loops from HIFI-predicted data for Fig. 4 comparisons.

6. Relate to Fig. 5, cross platform comparison. Both 6-cutter and 4-cutter data are available for GM12878 cells. Fig. 5 showing the results focusing on the difference between the two due to resolution difference. But I think it is also important to see consistency between the two. For example, the authors can convert high-resolution data into low-resolution data for consistency test. Actually this is relevant to my question 2. The author can use 4-base cutter (which is deeper) as test data to test how the downsampled 6-cutter works.

7. General comments. HIFI enhances raw matrix (with a global total reads bias correction). But whether there are also sources of biases that are not corrected (such as distance). Both true signal and bias can be enhanced in HIFI, which might limit the use of HIFI.

8. Technical question. there is an alpha parameter in MRF method. It is still not very clear to me how this is used, and how changing alpha may alter the results.

**Authors Response**

**Point-by-point responses to the reviewers’ comments:**

Reviewer #1: In this paper, the authors described a new method HIFI to estimate chromatin interaction from Hi-C data at fragment resolution. This work addresses an important research question. The proposed methodology is interesting. The authors performed comprehensive analysis to benchmark the performance of HIFI with existing methods. The paper is also well written. However, I still have a few comments and suggestions which may further improve the current manuscript. Here are my specific comments.

Major comments:

1. The major goal of this work is to perform Hi-C data analysis at fragment resolution. However, the authors only provided new estimate of fragment resolution interaction frequency. I feel this work is not complete. Without well-designed methods for fragment resolution Hi-C data normalization and significant chromatin interaction detection, it is difficult to interpret the output of HIFI. The potential impact of HIFI is limited. It would be a key contribution if the authors can provide a complete pipeline for Hi-C data analysis at fragment resolution for both 4 bp cutter and 6 bp cutter. In addition, for HindIII Hi-C data, Jin et al, Nature, 2013 study (PMID: 24141950) also did fragment resolution analysis. It is necessary to carefully compare HIFI with Jin et al's method.

*==> We thank the reviewer for pointing out this limitation. While we feel that a detailed analysis of the source and correction of biases in high-resolution HiC data is beyond the scope of this paper, we investigated the impact of different normalization algorithms on the ability of HIFI to recapitulate 5C data (see Suppl. Fig.7). Our results suggest that this factor has a limited impact, for reasons that are now explained in the Discussion section. However, we modified the HIFI software to accommodate user-defined normalization approaches, allowing users to use whichever normalization approach they want.*

2. Most results in this paper are based on Rao et al GM12878 data with 6bp cutter HindIII. However, the major deliverable in the Rao et al 2014 study is the in situ Hi-C experiment protocol and GM12878 data with 4 bp cutter MboI. GEO GSE6352 lists two replicates of GM12878 in situ Hi-C data (primary and replicate). The authors can use one as the training set, and the other as the testing set. More importantly, almost all recent Hi-C studies use in situ Hi-C with 4 bp cutter MboI or DpnII. Since the goal of this work is to estimate fragment resolution Hi-C contact frequency, I hope the author can present in situ Hi-C with 4 bp cutter in the main text, and move all results based on 6 bp cutter into supplementary.

*==> We followed the reviewer’s suggestion and applied HIFI to replicates of a deep 4-cutter HiC data set produced by Bonev et al (2017) for mouse ESC. We show that HIFI-MRF indeed yields significantly improved correlation between replicates (Suppl. Fig. 3), compared to standard fixed-binning approaches.*

*==> We elected to keep part of the focus of the manuscript on Rao’s HindIII data set rather than their 4-cutter MboI data set, for a variety of reasons. First, 4-cutter 5C/ChIA-PET data is not prevalent, which would prevent validation of our methodology. Second, Rao’s 4-cutter MboI Hi-C libraries are actually of lower quality than their HindIII library, and their sequencing depth would be insufficient to perform many of the analyses reported in the paper for HindIII (e.g. Figure 1). Finally, running time and memory requirements currently make it difficult to run the multiple whole-genome analyses at 4-cutter resolution (the analyses reported for MboI are limited to a 1Mb distance).*

3. In the section "validation again 5C data", the authors can use mESC Hi-C data from Bonev et al Cell 2017 paper (PMID: 29053968) and GM12878 in situ Hi-C data from Rao et al 2014. Those data are the deepest Hi-C data to date and with better quality than the Hi-C data currently used in this study.

*==> For this analysis, we elected to use HiC data obtained with the same restriction enzyme as the one used for the 5C data sets we are comparing against (HindIII). Although this meant using an older data set (Dixon 2012), this reduced potential biases due to the choice of restriction enzyme or conversion of resolutions between data sets, allowing us to focus the analysis on the relative merits of different HiC processing approaches.*

4. In addition to comparison with ChIA-PET data, I hope the authors can also compare HIFI-predicted Hi-C contact frequency with GM12878 HiChIP data (PMID: 28945252), which is the current state-of-the-art measurement of enhancer-promoter interactions.

*==> This is an excellent suggestion, and we added a new Supplementary Figure (Suppl. Fig. 10) to that effect, showing that HIFI performs comparably or better than existing approaches for highlighting HiChIP-detected contacts. The HiChIP dataset suggested by the reviewer contains only histone modification targets*

*(https://www.ncbi.nlm.nih.gov/geo/query/acc.cgi?acc=GSE101498), which may not be easy to interpret or able to be included within any of the current analyses. Instead, we performed our analysis against the cohesin GM12878 MboI HiChIP data set from Mumbach et al. 2016 (https://www.ncbi.nlm.nih.gov/pmc/articles/PMC5501173/pdf/nihms872416.pdf).*

*==> We also investigated comparisons against capture Hi-C (CHi-C) data. Validated promoter CHi-C contacts from Martin et al. (2015) proved to be too sparse and too biased toward extremely strong contacts to provide a useful basis for comparison.*

5. Page 9, line 52. The authors used a matrix-balancing algorithm to do fragment resolution bias normalization, which can be problematic. In literature, matrix-balancing algorithm has been applied to equally-binned Hi-C data, but not for fragment resolution Hi-C data. In addition, a recent study (Gilgenast and Phillips-Cremins, Cell Systems, 2019, PMID: 30904376) has shown that the equal visibility assumption does not apply to fragment resolution 5C data. So I would suspect that matrix-balancing algorithm is not appropriate for fragment resolution Hi-C data either. I hope the authors either develop a new normalization method, or use established methods in two previous studies (Yaffe and Tanay, Nature Genetics, 2011. PMID: 22001755 and Jin et al, Nature, 2013. PMID: 24141950)

*==> As mentioned earlier, we have now added results showing the impact of various normalization algorithms (Suppl. Fig. 7), which suggests that, at least when comparing against 5C data, it is difficult to conclude that one normalization approach is better than another. However, acknowledging that this may not always be the case, we added functionality to HIFI to allow users to provide their own normalization factors, and discuss this issue in the Discussion section.*

6. A key feature from high resolution Hi-C data is chromatin loop identified by HiCCUPS. It would be more convincing if HIFI-predicted chromatin interactions can help to improve statistical power of chromatin loop detection.

*==> We agree that it would be fantastic to be able to run HICCUPS on the HIFI-predicted IF matrix. We had detailed discussions with the authors of HICCUPS in this regard. Although HIFI’s output can be converted to the .hic format used by tools from the Juicebox package, HICCUPS (and algorithms implemented in Juicebox) expect/require fixed binned data. Currently, these algorithms are unable to run on restriction-fragment resolution data.*

7. I don't fully understand the method used in Figure 4. HIFI is used to estimate fragment resolution Hi-C contact frequency. Similar to HIFI, HiCPlus can be used to enhancer Hi-C data resolution. Different from HIFI and HiCPlus, HMRFBayes is a significant interaction caller. How did the authors obtain significant chromatin interactions from HIFI-predicted and HiCPlus-enhanced Hi-C data?

*==> Although each method differs in the meaning of the scores assigned to each pair of RFs, in all cases higher scores indicate higher confidence in the presence of an interaction. These scores can thus be used to obtain a ROC curve (separately for each range of distance), whose AUC is then reported. This measures the ability of a given approach to delineate functional interactions.*

Minor comments:

1. Page 3, line 61 - page 4, line 6. The authors mentioned that Hi-C data with billion reads still have very low average reads between each fragment end pairs. It would be more informative to show the average reads per fragment end pairs for each 1D genomic distance, and the percentage of fragment end pairs without any read for each 1D genomic distance. At fragment resolution, read count for pairs >1Mb or between different TADs is very sparse, therefore genome-wide average is not very informative.

*==> We have added a note to that effect at the beginning of the Results section.*

2. Page 3, line 40 - 44. I don't quite understand the approach 1. For fixed-binning approach, when combining consecutive fragments into bins, the unit of analysis is bin, not fragment. So one will only use the summation of all reads between bin pairs, without taking average for fragment pairs.

*==> Yes, we apologize, the wording was confusing. We have revised this description.*

3. Page 5, line 8. Typo: "HiPlus" should be "HiCPlus".

*==> Fixed*

4. Page 5, line 8 - line 11. The authors need to present the HiCPlus results. The current wording for the comparison with HiCPlus is very vague. It would be more convincing if the authors show concrete numerical results for HiCPlus.

*==> The results obtained with HiCPlus in this setting were nonsensical, which is why we elected not to report them. In-depth analysis and discussion with the HiCPlus authors identified the absence of bias correction as the key culprit. Since the analysis presented in that section are intrinsically non-bias corrected, it was impossible to use HiCPlus.*

5. Page 8, line 55. It takes around one hour per chromosome at HindIII resolution. What is the computational cost for analysis at MboI fragment resolution? Is it practical to apply HIFI to most in situ Hi-C data with 4 bp cutter?

*==> The running time for MboI analyses are indeed quite slow and memory intensive, which is why analyses were limited to the portion of the HiC matrix that includes contacts within a maximum distance of 1Mb. This is now clearly stated in the MboI section. These analyses completed in a few hours per chromosome and with ~100 Gb RAM.*

6. Page 10, line 25. How did the author choose the bandwidth parameter h?

*==> In Figure 1D and 1E, for each value along the x-axis, we report the result of the HIFI-KDE algorithm for the most favourable choice of h, where h ranges from 0.5 to 20 (see Suppl. Fig. 1C). In other words, different points along the HIFI-KDE curve were obtained with different values of h. The same holds for HIFI-AKDE, where multiple values of the MinimumCount parameter were tried, and the best result reported for each point along the x-axis. This arguably provides an unfair advantage to these two approaches, compared to HIFI-MRF. Despite this, HIFI-MRF outperforms them.*

7. Page 10, line 44. "MinimumCount" is a user-defined parameter. I hope the authors can provide some justification/guidance on how to choose this parameter in practice.

*==> We added text to that effect in Methods. Empirically, MinimumCount=100 works well on most data sets, irrespective of resolution.*

8. HIFI-MRF relies on some optimization algorithms. What is the reproducibility of HIFI-MRF output? Is it robust to initial values and random seeds?

*==> HIFI-MRF is actually a deterministic algorithm: the initial values are provided by the HIFI-AKDE algorithm (which is itself deterministic), and the optimization process is a deterministic gradient descent where matrix cells are always updated in the same order (top-to-bottom, left-to-right). We actually considered a version where cells are updated in random order. This converged to highly similar solutions, but the execution was slower due to poor cache use efficiency.*

Reviewer #2: Cameron et al. proposed a HIFI program for Hi-C data analysis. The idea is to impute deep Hi-C data at fragment resolution from sparse data. Conceptually it is possible to do this if data from neighboring fragments are taken. The paper compared several approaches and landed on the Markov random fields (MRF) method with the best outcome. The direct validation was done by comparing to two 5C data. Indirect validation was done by comparing to the ChIA-PET (CTCF and PolII) and E-P link (based on DHS correlation). The author also showed some examples using the enhanced data to analyze the features near TAD or sub-TAD boundaries.

Overall this is an interesting approach and good addition to the Hi-C analysis field. It is potentially useful when only low-depth Hi-C data is available. The computational method seems to be largely reasonable. On the other hand, from the point of view of users, this paper will benefit from providing more benchmarks and comparative analyses. A little restructuring of the results can be helpful to readers, too. Below are my comments.

1. The authors listed 4 HIFI method but starting from Fig. 2, Fixed bin approaches are quite obvious. HIFI-KDE or HIFI-AKDE are almost never mentioned after Fig. 1. The authors clearly prefer HIFI-MRF. I suggest the authors to move some results to supp and keep the readers focusing on HIFI-MRF.

*==> While HIFI-MRF is indeed clearly superior to the other three approaches, we feel that it is important to introduce all three other approaches, for the following reasons. First, HIFI-MRF is a somewhat complicated approach, and it is our belief that one should not introduce a complex approach without demonstrating that it outperforms simpler ones. HIFI-KDE and HIFI-AKDE were thus introduced to provide a meaningful benchmark for HIFI-MRF. Second, HIFI-MRF actually relies on HIFI-AKDE for its initialization, and thus HIFI-AKDE needs to be introduced.*

2. Relate to Fig. 1, choice of test data. For cross validation, the authors reserve 20% data as test set and downsampled the rest data at different level and computed the correlation between HIFI-predicted data to the test data. I feel a little confused why only choosing 20% as test data. For example, the HIFI predicted data from 80% data will be cross-validated to un-enhanced low depth data, which can be misleading.

*==> We understand that this procedure can feel counter-intuitive. Of course, the test IF matrix obtained from only 20% of the reads pairs is not itself the true IF matrix. However, because this test set represents a data set that is sampled from the true IF independently from the training set, the predictor that maximizes the likelihood of this test set (or equivalently minimizes the sum-of-squared errors) is also the predictor that indeed has the highest expected accuracy. The validity of the argument is actually independent of the actual size of the test set; only the variance of conclusions depend on it, and at 20% = 152 Million read pairs, this variance is minuscule.*

*==> We thus elected to preserve most of the reads for the different subsampled training sets, in order to be best able to monitor the performance of the different approaches on training sets ranging from very small (6 Million reads) to very large (608 Million reads). However, in preliminary experiments not reported here, we obtained obtained nearly identical results from a train/test split of 50-50.*

3. Reproducibility. Published deep Hi-C data are usually from multiple biological replicates. Can the authors assess how reproducible their result is from different biological replicates? E.g. showing the results on the two 5C locus.

*==> As mentioned above in our response to a similar comment from reviewer #1:*

*We followed the reviewers’ suggestions and applied HIFI to replicates of a deep 4-cutter Hi-C data set produced by Bonev et al (2017) for mouse ESC. We show that HIFI-MRF indeed yields significantly improved correlation between replicates (Suppl. Fig. 3), compared to standard fixed-binning approaches.*

4. Lower limit of method. The depth of data obviously cannot be too low. As a potential user, I will be interested in knowing about the limitations of the method. What is the low limit of sequencing depth? Results from down-sampled data on the 5C results will be helpful.

*==>This is a very interesting question and indeed the analyses presented in Figure 4 and Suppl. Fig. 8-10 address this point. Yet this deserves more discussion, and we have added a paragraph to that effect in Discussion. Although we analyzed the impact of Hi-C sequencing depth on the correlation with 5C data, the result was not very informative, showing a slow decline as one lowers depth with no particular break point of interest, very similarly to what is seen in Figure 1D.*

5. Relate to Fig. 4, loop calling method? I did not find the detailed method on how the authors call loops from HIFI-predicted data for Fig. 4 comparisons.

*==> The analysis reported here is not based on a sophisticated loop-calling method such as HICCUPS, but instead simply assesses the extent to which HIFI’s predicted IF values can be used directly as a predictor of ChIA-PET interactions. For each range of genomic distances, contacts are ranked based on their predicted IF, and the AUC of the resulting ROC curve is computed and reported.*

6. Relate to Fig. 5, cross platform comparison. Both 6-cutter and 4-cutter data are available for GM12878 cells. Fig. 5 showing the results focusing on the difference between the two due to resolution difference. But I think it is also important to see consistency between the two. For example, the authors can convert high-resolution data into low-resolution data for consistency test. Actually this is relevant to my question 2. The author can use 4-base cutter (which is deeper) as test data to test how the downsampled 6-cutter works.

*==> The reviewer’s suggestion is quite interesting and we have investigated this direction. We converted Rao’s MboI data to HindIII resolution, and used it as a reference against which we compared all HindIII-based prediction approaches (fixed binning and all the HIFI variants). The results are somewhat surprising: the sum of squared errors obtained by all approaches are approximately 10 times worse than they are in our cross-validation study (Figure 1D), suggesting that Rao’s MboI data is actually a poor approximation to their HindIII data. Compared against this benchmark, the performance of fixed binning (b=16), HIFI-KDE, HIFI-AKDE, and HIFI-MRF approaches are all comparable, but we elected not to report those results in the paper, as we don’t believe they represents a meaningful evaluation of the methods themselves. Rather, this speaks to the fact that, at high resolution, the choice of restriction enzyme has a significant impact on the results. However, investigating this interesting question will be done in a separate paper.*

7. General comments. HIFI enhances raw matrix (with a global total reads bias correction). But whether there are also sources of biases that are not corrected (such as distance). Both true signal and bias can be enhanced in HIFI, which might limit the use of HIFI.

*==> The reviewer is correct that the goal of HIFI is not primarily to detect or correct biases, but rather to provide raw interaction frequency estimates of the highest possible accuracy at high resolution. Our new analyses against 5C data (Suppl. Fig. 7) suggest that differences between the results of different types of bias correction algorithms may not be dramatic in the context of HIFI. Yet, HIFI will benefit from future improvements to RF-resolution bias correction, and indeed we have added new functionality to the HIFI package to more flexibly accommodate a variety of bias correction approaches.*

8. Technical question. there is an alpha parameter in MRF method. It is still not very clear to me how this is used, and how changing alpha may alter the results.

*==> Indeed, the reviewer has a sharp eye! Alpha is the only hyper-parameter in HIFI-MRF, and its role is to quantify the strength of the dependency between the IF of neighboring cells. A small value of alpha results in a strong dependency between neighboring cells, and hence a very smooth predicted IF matrix. A larger value results a “bumpier” matrix. Alpha was estimated using maximum likelihood on the Rao. et al. 2014 data set, i.e. by assessing the likelihood of the left-out data for values of alpha ranging from 0.01 to 1. Remarkably, although alpha=0.2 produced the best results, the predicted IF matrices obtained using alpha ranging from 0.1 to 0.25 were very similar. This is due to the fact that choosing alpha effectively determines the prior on the full IF matrix, but this prior is weak compared to the data itself, and hence the predicted IF matrices are mostly driven by the data rather than the prior.*

**Second round of review**

**Reviewer 1**

The authors did good job in revision and the manuscript has been improved significantly. However, several major comments are not fully addressed.

1. My previous major comment #1. As a method paper in the high profile journal Genome Biology, I feel "a detailed analysis of the source and correction of biases in high-resolution Hi-C data" is with the scope in this work, and should be fully elaborated. I would expect the authors to provide a complete pipeline for fragment resolution Hi-C data analysis, including both data normalization and significant chromatin interaction detection. In addition, I did not find the comparison between HIFI and Jin et al's method (PMID: 24141950) in the revised manuscript.

2. My previous major comment #6. I hope to the authors can clearly demonstrate that whether HIFI-predicted chromatin interactions can better detect chromatin loops. I understand that HiCCUPS only takes fixed binned data as input. The authors can modify the local background model used in HiCCUPS, and call chromatin loops in fragment resolution HIFI-predicted Hi-C data.

3. My previous major comment #7. In the response letter, the authors said "in all cases higher scores indicate higher confidence in the presence of an interaction", which is not accurate. The presence of interaction depends on specific background model, not only the predicted interaction frequency/score. Without a specific background model and the algorithm for detecting significant chromatin interactions at fragment resolution, it is hard to interpret Figure 4.

**Reviewer 2**

The authors answered most of my concerns. However, two important points coming from the rebuttal were not included in the paper, which I think is necessary to make a solid paper.

My question #5. The authors explained that they used a simple method directly call loops based on interaction frequency. I am ok with this explanation but this should be included in the text or figure legend.

The authors’ answer to my question #6 proves my concern in question #7. Hi-C data with different enzymes have different bias structure. HIFI enhance raw reads, therefore the same error structure is also enhanced. This is why the MSE between 4-cutter and 6-cutter is much higher. I strongly believe this should be clearly pointed out here, instead of a separate paper. I agree with the authors that this is not the problem of their method, therefore there is no shame to show this.

**Authors Response**

**Point-by-point responses to the reviewers’ comments:**

Reviewer #1: The authors did good job in revision and the manuscript has been improved significantly. However, several major comments are not fully addressed.

1. My previous major comment #1. As a method paper in the high profile journal Genome Biology, I feel "a detailed analysis of the source and correction of biases in high-resolution Hi-C data" is with the scope in this work, and should be fully elaborated. I would expect the authors to provide a complete pipeline for fragment resolution Hi-C data analysis, including both data normalization and significant chromatin interaction detection.

Although the editor suggested that providing such a complete pipeline may not be absolutely required, we agree that providing support to normalization is needed to ensure adoption. Because existing HiC software for normalization, loop/TAD/compartment calling, and visualization all operate at fixed resolution, we added to HIFI the functionality needed for conversion from restriction fragment resolution to fixed resolution. This enables HIFI to be plugged in any existing HiC analysis pipeline (e.g. Juicer and its excellent normalization and visualization functionality), without the need to reinvent the wheel. We also introduced a simple HICCUPS-inspired loop-calling program, which operates on HIFI-processed restriction-fragment resolution matrices and show in the manuscript (Supplemental Figure 11) that it performs well in comparison to HICCUPS.

In addition, I did not find the comparison between HIFI and Jin et al's method (PMID: 24141950) in the revised manuscript.

*Indeed, we were unable to perform an empirical comparison to the work of Jin et al., as no implementation of their approach is available. Since the model, proposed by Jin et al. is not a deterministic model and weight/coefficients must be learned. There is no guarantee that we would recapitulate their work.*

2. My previous major comment #6. I hope to the authors can clearly demonstrate that whether HIFI-predicted chromatin interactions can better detect chromatin loops. I understand that HiCCUPS only takes fixed binned data as input. The authors can modify the local background model used in HiCCUPS, and call chromatin loops in fragment resolution HIFI-predicted Hi-C data.

*While the primary goal of HIFI is not to identify loops but rather to accurately estimate entire IF matrices, we agree there is an opportunity to build upon HIFI-processed matrices to identify loops with great accuracy. We thus followed the reviewer’s suggestion and developed a HiCCUPS-inspired loop-calling program, which is shown to perform very well. This is now part of the HIFI distribution. Please see additions to Supplemental Figure 11 and Methods.*

3. My previous major comment #7. In the response letter, the authors said "in all cases higher scores indicate higher confidence in the presence of an interaction", which is not accurate. The presence of interaction depends on specific background model, not only the predicted interaction frequency/score. Without a specific background model and the algorithm for detecting significant chromatin interactions at fragment resolution, it is hard to interpret Figure 4.

*We hope that our response to the previous point and the development of a loop-calling module in HIFI addresses this question. With regards to background models, our loop calling module uses an approach similar to HiCCUPS, where IF values in a small region of the matrix (candidate loop) are contrasted against the surrounding IF values (either all around (donut), horizontally, vertically, or to the bottom left).*

*We elected to also keep the original analysis presented in Figure 4, where IF values are directly used as a predictor of functional contact. Importantly, we have clarified that since different predictors are trained and used separately for each distance bin (diagonal of the Hi-C matrix), this effectively amounts to using a simple distance-based background model. Indeed, the AUC values reported in the Figure are effectively a measure of the association between the ranking of an IF value with respect to the diagonal it belongs to, and the presence of externally determined contacts (e.g. ChIA-PET).*

Reviewer #2: The authors answered most of my concerns. However, two important points coming from the rebuttal were not included in the paper, which I think is necessary to make a solid paper.

My question #5. The authors explained that they used a simple method directly call loops based on interaction frequency. I am ok with this explanation but this should be included in the text or figure legend.

*We have now added an entirely new loop calling module similar to HiCCUPS and show that it performs very well when applied to HIFI-processed HiC matrices Please see additions to Methods.*

The authors’ answer to my question #6 proves my concern in question #7. Hi-C data with different enzymes have different bias structure. HIFI enhance raw reads, therefore the same error structure is also enhanced. This is why the MSE between 4-cutter and 6-cutter is much higher. I strongly believe this should be clearly pointed out here, instead of a separate paper. I agree with the authors that this is not the problem of their method, therefore there is no shame to show this.

*We added a paragraph to this effect in the Discussion section.*

**Third round of review**

**Reviewer 1**

The authors did excellent job in the revision and fully addressed my previous comments and suggestions. The paper has been significantly improved. I think the current version is ready for publication.
